# X-linked myotubular myopathy is associated with epigenetic alterations and is ameliorated by HDAC inhibition

**DOI:** 10.1007/s00401-022-02468-7

**Published:** 2022-07-17

**Authors:** Jonathan R. Volpatti, Mehdi M. Ghahramani-Seno, Mélanie Mansat, Nesrin Sabha, Ege Sarikaya, Sarah J. Goodman, Eric Chater-Diehl, Alper Celik, Emanuela Pannia, Carine Froment, Lucie Combes-Soia, Nika Maani, Kyoko E. Yuki, Gaëtan Chicanne, Liis Uusküla-Reimand, Simon Monis, Sana Akhtar Alvi, Casie A. Genetti, Bernard Payrastre, Alan H. Beggs, Carsten G. Bonnemann, Francesco Muntoni, Michael D. Wilson, Rosanna Weksberg, Julien Viaud, James J. Dowling

**Affiliations:** 1 Department of Molecular Genetics, University of Toronto, Toronto, ON M5S 1A1, Canada; 2 Program for Genetics and Genome Biology, The Hospital for Sick Children, 555 University Ave, Toronto, ON M5G 0A4, Canada; 3 Institute of Cardiovascular and Metabolic Diseases (I2MC), INSERM, UMR-S U1297 and University of Toulouse III, CHU-Rangueil, Toulouse, France; 4 Institute of Medical Sciences, University of Toronto, Toronto, ON M5S 1A1, Canada; 5 Institut de Pharmacologie Et Biologie Structurale (IPBS), Université de Toulouse, CNRS, UPS, Toulouse, France; 6 Program for Genetics and Genome Biology, The Hospital for Sick Children, Toronto, ON M5G 0A4, Canada; 7 Laboratoire d’Hématologie, Centre Hospitalier Universitaire (CHU) de Toulouse, Toulouse Cedex, France; 8 Division of Genetics and Genomics, The Manton Center for Orphan Disease Research, Boston Children’s Hospital, Harvard Medical School, Boston, MA, USA; 9 Neuromuscular and Neurogenetic Disorders of Childhood Section, NINDS, NIH, Bethesda, MD, USA; 10 Dubowitz Neuromuscular Centre, NIHR Great Ormond Street Hospital Biomedical Research Centre, Great Ormond Street Institute of Child Health, University College London, London, UK; 11 Department of Paediatrics, University of Toronto, Toronto, ON M5S 1A1, Canada; 12 Division of Clinical and Metabolic Genetics, The Hospital for Sick Children, Toronto, ON M5G 0A4, Canada; 13 Division of Neurology, The Hospital for Sick Children, Toronto, ON M5G 0A4, Canada

**Keywords:** X-linked myotubular myopathy, Congenital myopathies, Epigenetics, Drug discovery

## Abstract

X-linked myotubular myopathy (XLMTM) is a fatal neuromuscular disorder caused by loss of function mutations in *MTM1*. At present, there are no directed therapies for XLMTM, and incomplete understanding of disease pathomechanisms. To address these knowledge gaps, we performed a drug screen in *mtm1* mutant zebrafish and identified four positive hits, including valproic acid, which functions as a potent suppressor of the *mtm1* zebrafish phenotype via HDAC inhibition. We translated these findings to a mouse XLMTM model, and showed that valproic acid ameliorates the murine phenotype. These observations led us to interrogate the epigenome in *Mtm1* knockout mice; we found increased DNA methylation, which is normalized with valproic acid, and likely mediated through aberrant 1-carbon metabolism. Finally, we made the unexpected observation that XLMTM patients share a distinct DNA methylation signature, suggesting that epigenetic alteration is a conserved disease feature amenable to therapeutic intervention.

## Introduction

X-linked myotubular myopathy (XLMTM) is a rare neuromuscular disorder caused by loss-of-function mutation in the *MTM1* gene [38]. Male patients present at or around the time of birth with hypotonia and weakness [40]. 25–50% of affected individuals die in the first year of life; survivors have significant technology dependences, with the majority (80%) requiring wheelchair, ventilator, and feeding tube support[2, 3, 7]. There are currently no approved treatments for this devastating condition.

*MTM1* encodes a lipid phosphatase, myotubularin (MTM1), that dephosphorylates phosphatidylinositol (PtdIns)-3-phosphate (PtdIns3P) and PtdIns(3,5)P_2_ [8, 58]. MTM1 is a resident of the endosome [62], where it is involved in a phosphoinositide conversion mechanism from PtdIns3P to PtdIns4P that allows cargo to recycle from endosomal compartments to the plasma membrane [37]. Loss of MTM1 results in dramatic alteration of skeletal muscle cytoarchitecture, including reduced myofiber size, increased nuclear centralization, and disorganization of the sarco-tubular network [39]. It is also associated with alteration of several cellular pathways, including autophagy [1, 25, 28], proteasomal turnover [1, 30], mitochondrial oxidative phosphorylation [34], and integrin trafficking [48]. While plausible that regulation of endomembrane phosphoinositides and membrane traffic by MTM1 lies upstream of most cellular and molecular defects observed in XLMTM, there is incomplete understanding of the interplay between normal MTM1 function, loss of MTM1 expression, and disease pathogenesis.

There is a great unmet need for therapies for XLMTM. AAV8-mediated gene replacement therapy has demonstrated efficacy in pre-clinical models of XLMTM [17], and is currently under evaluation in clinical trial (NCT03199469). Additional therapeutic strategies include reduction of dynamin-2 (DNM2), achieved either with antisense oligonucleotides to the *DNM2* gene or tamoxifen treatment [31, 42, 57], both of which are also in clinical trial (NCT04743557, NCT04033159, and NCT04915846), and inhibition of the class IIB PI 3-kinase (PIK3C2B) [53], which acts reciprocally to MTM1 to regulate PtdIns3P and PtdIns4P in preclinical models.

To identify new therapeutic strategies and advance understanding of disease pathomechanisms, we performed a large scale drug screen in a zebrafish XLMTM model. In this screen, we identified valproic acid (VPA) as a dose-dependent modifier of the zebrafish phenotype, and demonstrated that VPA acts primarily via its action as a histone deacetylase inhibitor (HDACi). We orthogonally validated the efficacy of VPA in a mouse XLMTM model and in *Mtm1* deficient C2C12 cells. We show that VPA acts in part via modification of the XLMTM transcriptome and also via correction of defective integrin trafficking. Lastly, we demonstrate epigenetic abnormalities in XLMTM, changes likely driven by altered 1-carbon metabolism and that are partially improved by VPA treatment. Importantly, aberrant DNA methylation was identified in XLMTM patient samples, suggesting a key role for epigenetic regulation in XLMTM and supporting the translatability of our pre-clinical findings.

## Materials and methods

### Animal handling and welfare

Mice were housed at the Toronto Centre for Phenogenomics (TCP). The *Mtm1* KO line, originally generated at the IGBMC (Strasbourg, France, kind gift of Jocelyn Laporte) [13], was rederived onto the C57BL/6 J background at TCP. All mice were housed 1–5 per cage with standard bedding on a 12-h daylight cycle and had unlimited access to water and food (Teklad Rodent Diet #2918, Envigo). PCR was used to detect the deletion in exon 4 of *Mtm1* KO mice. All zebrafish studies were conducted in the PGCRL at the Hospital for Sick Children (Toronto). Mutants were generated and maintained on both AB and TU wild-type (WT) strains. Adults were maintained at 28.5 °C on a 14/10-h light/dark cycle. Genotyping was performed using genomic DNA from adult fins or embryo. All mice and zebrafish studies were conducted in compliance with the Animals for Research Act of Ontario and the Guidelines of the Canadian Council on Animal Care (AUP#22–0255H; AUP#41617).

### Generating zebrafish *mtm1* mutants

A new *mtm1* allele was generated by CRISPR/Cas9 mutagenesis following established methods [36, 60] using a single guide RNA (sgRNA) targeting exon 11 of *mtm1* that encodes the phosphatase active site (CSDGWDR) [20]. A 12 bp in-frame deletion (c.1283_1294delGCA GTG ATG GCT) was identified and predicted to result in loss of CSDG of the active site motif (p.(Cys406_Gly409del)). Sequence analysis was assisted by TIDE and confirmed manually. In-cross progeny from the F_3_ generation (or greater) were characterized in this study.

### Zebrafish phenotypic drug screen

A library of 1280 clinical trial stage chemicals (US Drug Collection, MicroSource) was screened for chemicals that could suppress fin fold degeneration in *mtm1* mutants. All library stocks were diluted to a 10 μM screening concentration. Plates were incubated at 28.5 °C until 4 dpf to assess fin degeneration. Follow-up screens were performed in 24-well plates and 1 mL egg water plus chemical to eliminate false positives and identify true hits. Chemicals that showed a strong dose response were considered chemical suppressors of *mtm1* fin degeneration.

### Zebrafish chemical assays

Chemical stocks were prepared in DMSO (unless otherwise noted) and added to egg water (no methylene blue) at 0.1–0.5% of the final volume to prepare working concentrations. Equal volumes of vehicle solvent were used in all conditions for a single assay. Dishes were incubated at 28.5 °C until the assay date. For all zebrafish assays, larvae were evenly distributed from one pair of parents across all conditions and any dysmorphic embryos were removed prior to plating to minimize clutch-to-clutch variability.

### Fin degeneration assay

To assess fin degeneration in *mtm1* mutants, between 100 and 250 embryos (1 dpf) from single-pair *mtm1*^+/zf711^ adult in-crosses were distributed into sterile 10 cm tissue culture dishes (Falcon) containing 40 mL of egg water plus chemical. At 4 dpf, larvae were anesthetized with tricaine and fin fold morphology was scored on a 4-point ordinal scale to rank the severity of fin degeneration as follows: WT-like (0), mild (1), moderate (2), and severe (3) [53]. For imaging, larvae were mounted in 3% methylcellulose on glass slides and bright-field images were taken with a light microscope (Olympus BX43).

### Maximum likelihood phylogenetic tree of HDACs

Primary sequences for 33 full-length histone deacetylase (HDAC) proteins from human, mouse, and zebrafish were obtained from UniProt database using their canonical isoforms, with the exception of human HDAC9 for which the N-terminal splice variant (isoform 3; a.k.a. MITR) was used (UniProt accession numbers: Q13547, O09106, F1R653, Q92769, P70288, O15379, O88895, Q803C3, P56524, Q6NZM9, Q08BS8, Q9UQL6, Q9Z2V6, E7FH14, Q9UBN7, Q9Z2V5, F8W4B7, Q8WUI4, Q8C2B3, A0A0G2KIA0, E7FAL6, Q9BY41, Q8VH37, Q7SXM0, Q9UKV0 [isoform 3], Q99N13, Q6PBI4, Q969S8, Q6P3E7, F1QCV2, Q96DB2, Q91WA3, F1QED3). Multiple sequences were aligned, and maximum likelihood-based inference performed with MEGA X software.

### Chemical-genetic fingerprint analysis

VPA was tested along with structurally dissimilar HDAC inhibitors and other chemical suppressors vs chemical enhancers of *mtm1* fin degeneration. 100 larvae per chemical were tested using our fin degeneration assay. Identified enhancers included tunicamycin (Tm), brefeldin A (BFA), dithiothreitol (DTT), bortezomib, 2-hexyl-1-decanol (HD), thimerosal (THM). Identified suppressors included cycloheximide (identified in the screen in this paper), wortmannin [53], dynasore, endosidin-2, and δ-hexachlorocyclohexane (δ-lindane). The mean rank scores for each chemical-chemical combination were determined (Kruskal–Wallis test with Dunn’s post-test; GraphPad Prism) and normalized to the mean rank score for DMSO-treated larvae. These data were transformed, and hierarchically clustered in Cluster 3.0 as previously described [29].

### Morpholino knockdown

Embryos resulting from *mtm1*^+/zf711^ in-crosses were used for injections. Between 1 and 6 nL of MO was delivered into the yolks of 1- to 4-cell stage embryos. We co-injected p53 morpholino (MO) at equimolar concentration with previously published MOs targeting *hdac1* (0.015 pmol/ ~ 125 pg), *hdac3* (1.2 pmol/ ~ 10 ng), *hdac6* (0.225 pmol/ ~ 1.8 ng), or *hdac8* (0.25 pmol/ ~ 2 ng). p53 was injected at half the molar concentration of *hdac3* MO. Controls were co-injected with p53 MO and a standard control MO designed to a random sequence that is not found in the zebrafish genome (Gene Tools, LLC).

### Photoactivation of motor behaviour assay

**Fig. 1:**
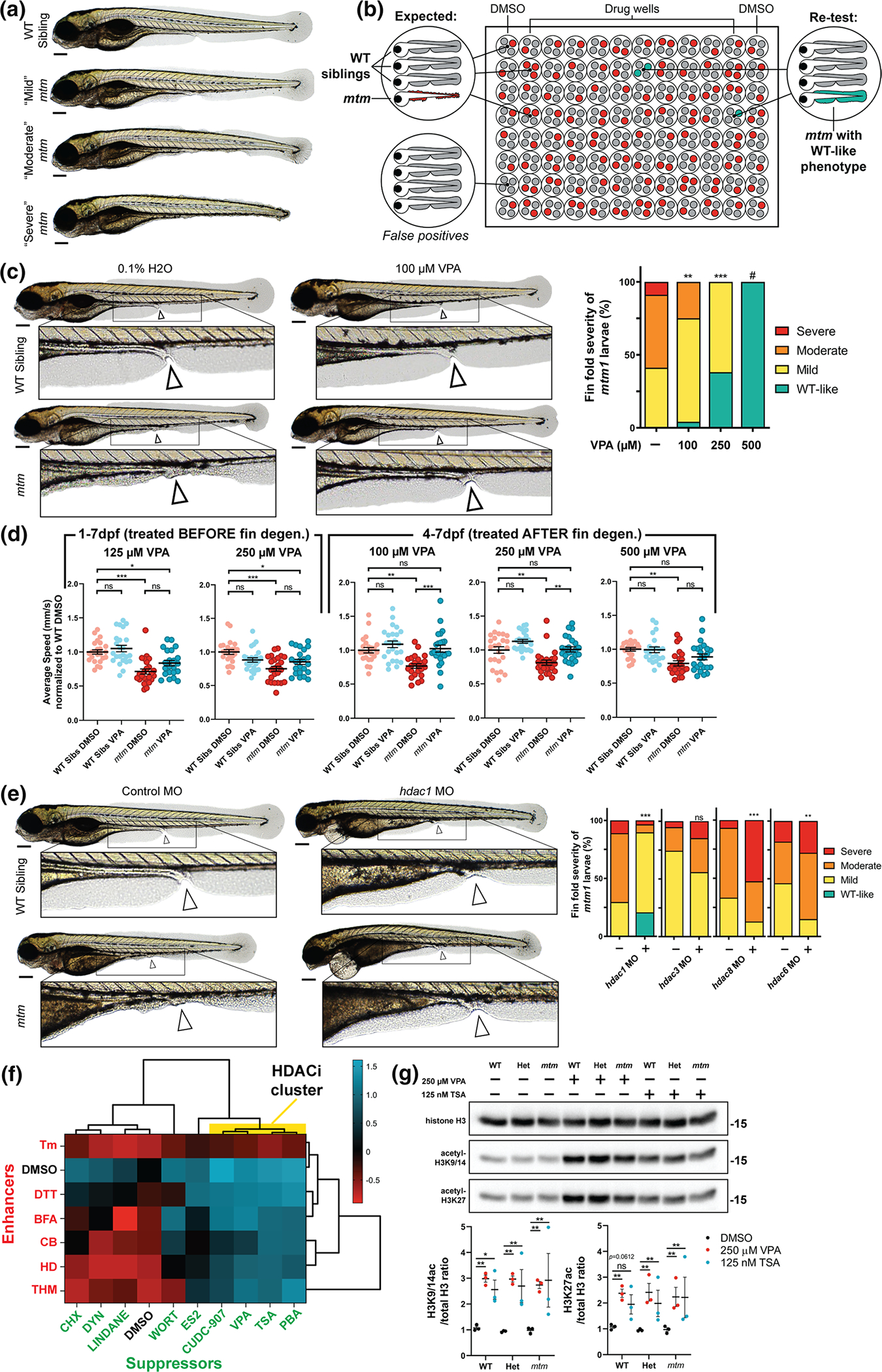
Phenotypic screen of *mtm* mutants identifies sodium valproate (VPA) as a suppressor of fin degeneration. **a** *mtm1*^Δ8/Δ8^ mutants (*mtm*) exhibit a spectrum of larval fin fold degeneration at 4 dpf, ranging from mild to severe. **b** Schematic of the unbiased screen designed to identify chemicals that suppress *mtm* fin degeneration. In total, 1280 chemicals were screened. **c** VPA was identified as a suppressor in the screen when larvae were exposed from 1 to 4 dpf. Escalating VPA concentrations show increasing effectiveness in suppressing fin degeneration. (Kruskal–Wallis test with Dunn’s post-test; Mean ± SEM; *n* = 68,24,21,0). #Note: no mutants were identified at 500 μM concentration; however there is considerable developmental toxicity at this concentration. 1 mM VPA is lethal to all fish exposed from 1 to 4 dpf. **d** VPA exposure promotes improvement in the motor activity of *mtm* mutants (2-way ANOVA with Tukey’s post-test; *n* = 22–24 larvae each group). To account for a potential advantage of having intact fin folds due to rescue by VPA, motor behavior was assessed after fins degenerated using severe *mtm* larvae only. **e** Morpholino knockdown of *hdac1* (zebrafish ortholog of human HDAC1/2) suppresses fin degeneration in *mtm* mutants. However, knockdown of class I *hdac3* and *hdac8*, or class II *hdac6*, failed to suppress the phenotype (two-tailed Mann–Whitney test; *n* = 29–90). **f** Chemical-chemical fingerprint reveals a common mode of action between VPA and HDAC inhibitors PBA, TSA (trichostatin A), and CUDC-907 in suppressing the fin phenotype (HDACi cluster in yellow) when combined with “enhancer” chemicals that worsen *mtm* fin degeneration. Other suppressors do not overcome the effects of enhancers, suggesting different mechanisms of suppression are involved. **g** Western blot reveals that larvae exposed to VPA and TSA have elevated levels of acetylated histones, consistent with HDAC inhibitor activity. Statistics: *p < 0.05, **p < 0.01, ***p < 0.001

Up to 50 larvae from single-pair *mtm1*^+/ZF711^ adult in-crosses were distributed tissue culture dishes containing 10 mL of egg water plus chemical. All assays were performed at 7 dpf using ZebraBox platform (ViewPoint) and 10 μM optovin analog 6b8 as previously described [53]. Larvae were either exposed to chemicals at 2.5 dpf (pre-symptomatic assay) or maintained in system water (post-symptomatic assay) before they were identified and separated by their fin morphology at 4 dpf. Larvae were returned to freshly prepared chemical plates or system water until the 7 dpf when they were assayed. Only *mtm1* mutants with “severe” fin degeneration (see Fig. 1) were used to minimize potential effects arising from remaining fin folds on swimming speed. We estimated chemical-genetic interactions based on an unexpected change in the difference between WT and *mtm1* following chemical treatment following a previously described protocol [63].

### Oil red O staining

At 7dpf, chemically treated larvae were fixed in 4% PFA overnight and depigmented using a bleach solution (1.5% H2O2 and 1% KOH) for 20 min at room temperature then rinsed with PBS. Larvae were transferred to 1.5 mL Eppendorf tubes and rinsed three times for 5 min with 1X PBS/0.5% Tween-20 (PBS-Tween). Larvae were then stained with 300 μL of 0.5% Oil Red O in 100% isopropyl alcohol and 200 μL of distilled water for 15 min.

### Quantification of neutrophils and macrophages in zebrafish trunks

At 1dpf, embryos from crosses between *mtm1*^+/zf711^ carriers with either *mtm1*^+/zf711^; Tg(mpeg1:eGFP) or *mtm1*^+/zf711^; Tg(mpx:GFP) adults were treated with either 0.1% DMSO, 250 μM VPA, or 125 nM trichostatin A. At 4 dpf, live embryos were anaesthetized were imaged using a Nikon A1 confocal microscope with 20 × objective.

### RNA sequencing (zebrafish)

At 1 dpf, embryos from single-pair in-crossings of *mtm1*^+/zf711^ adults were divided equally into three chemical treatment groups: 0.5% DMSO, 250 μM sodium valproate (VPA), or 125 nM trichostatin A. At 4 dpf, larvae were anaesthetized with tricaine and sorted by their fin degeneration phenotype (see above). For tissue collection, the posterior section of larvae, which excluded the heart, liver, brain, and eyes and was enriched in skeletal muscle and fin fold tissues was dissected and snap frozen on dry ice. A total of *n* = 6–10 larvae per pool per condition with five biological replicates were used for RNA extraction using Qiagen RNeasy kit. RNA concentration and purity were quantified using Qubit 4.0 (Invitrogen) and Bioanalyzer 2100 (Agilent). The anterior section (the head) was used to confirm genotype (either *mtm1*^+/+^ [WT] or *mtm1*^+/zf711^ [HET]). RNA libraries were generated using the QuantSeq 3’ mRNA sequencing kit (Lexogen) automated on an NGS workstation (Agilent) at The Centre for Applied Genomics (TCAG, Toronto, Canada). RNA sequencing was performed using a single end 50 bp extended to 68 bp flowcell on an Illumina HiSeq2500. The dataset was aligned to the zebrafish genome (GRCz9) using STAR, read quality assessed with FastQC, and adaptors trimmed using Cutadapt. Relative gene expression was estimated between groups using DESeq2. GO term enrichment of differentially expressed genes (DEGs) was analyzed with Metascape. Human orthologs of zebrafish DEGs were retrieved using g:Profiler (BIIT Group, University of Tartu) and used for Connectivity Map analysis.

### Drug treatments in mice

For all treatments, mice were dosed twice daily starting at 21 days until endpoint (defined as having lost > 10% body weight) via intraperitoneal injections. Treatment groups included either (1) VPA or PBS vehicle (240 mg/kg body weight), or (2) TSA or 0.5% DMSO vehicle (0.6 mg/kg body weight). Body weights were monitored two to three times per week. All experiments were performed in a single-blinded fashion.

### Mouse skeletal muscle histology and immunostaining

Frozen mouse tibialis anterior (TA) muscle from 35 day old mice was cut into 8-mm cross-sections and stained with Mayer’s haematoxylin and eosin (H&E). The number of centrally nucleated fibres and myofibers size quantification was performed manually at 40X magnification with an Olympus BX43 light microscope. Immunofluorescence (IF) was conducted as previously described [43]. For fibre typing, double IF was performed using antibodies against dystrophin (Abcam #ab15277, 1/500) in combination with either myosin heavy chain type 1 (type I, slow, Sigma #M8421, 1/50) or myosin heavy chain type 2b (fast, DSHB, #BF-F3,1/50). Myofiber size measurements were reported as minimal Feret’s diameter as previously described [53]. In addition, a Fiji macro (see Supplementary Materials Table) was written to automatically detect and quantify cross-sectional area of fibres outlined by IF staining to dystrophin (Supplementary Fig. 3). For p-SMAD immunofluorescence, p-SMAD2/SMAD3 (Thr8) was used (ThermoFisher, cat # PA5–99378, 1/100) and the fraction of positive p-SMAD 2/3 nuclei/total nuclei were determined using ImageJ.

### Transmission electron microscopy (TEM)

TA muscles from 35 day old mice were prepared for TEM as previously described [45]. Further processing was performed by the Advanced Bioimaging Center (The Hospital for Sick Children, Toronto, Canada).

### RNA sequencing (mice)

RNA extraction, library preparation, and RNA sequencing were performed as previously described [42]. Sequencing was done at TCAG using Illumina HiSeq 2500. Paired end read sequencing was performed to achieve 62–67 million reads per sample. Reads were aligned to the mouse genome (GRCm38/mm10) using STAR (v 2.6.1) with basic two pass mode. The gene level quantitation was performed using RSEM (v 1.2.22). Expected counts for each gene/transcript were then rounded to the nearest integer and these values were used for differential expression with DESeq2 package (v 1.22.2) within the R programming environment (v 3.5). GO term enrichment of DEGs was performed with Metascape and GSEA. Human orthologs of mouse DEGs were retrieved using g:Profiler (BIIT Group, University of Tartu) and used for GO term enrichment with GSEA as well as for Connectivity Map analysis.

### Proteomic analysis

Quadriceps muscle (*n* = 3 biological replicates for each treatment group) from 35 day old mice were lysed in RIPA buffer with inhibitors and 0.5% SDS. Proteomics analysis was conducted by the Network Biology Collaborative Centre (NBCC) at Lunenfeld-Tanenbaum Research Institute (Toronto, ON). A total of 50 μg of protein processed using S-traps micro spin columns (ProtiFi) per manufacturer’s protocols and digested with 2 μg of trypsin for 1 h at 47 °C. Tandem mass tag (TMT) labelling was performed to quantify all samples. Approximately 10 μg of each sample was labelled with 80 μg of its respective TMTpro 16plex label (ThermoFisher) per manufacturer protocols. Samples were lyophilized and 1/20th of each labelled sample was combined. High pH fractionation was performed and 3/4 of each labelled sample was combined for ~ 96 μg and fractionated with Pierce High pH fractionation kit (Cat#: 84868) per kit instructions. Trapped and trypsin-digested peptides were acquired on a 90 min gradient using an Orbitrap Fusion Lumos Tribrid mass spectrometer. Data was searched with SequestHT using the UP000000589 proteome (*Mus musculus*) generated by Uniprot, post-processed with Percolator, and analyzed using Proteome Discoverer 2.2 (Thermo Scientific). Metascape was used for GO term enrichment analysis of differentially expressed proteins (log2FC > 0.585, *p* value < 0.05).

### DNA 5-methylcytosine (5mC) quantification

DNA was extracted from TA of 35 day old mice using phenol–chloroform extraction method. Global DNA 5-mC was quantified using a MethylFlash Methylated DNA Quantification Kit (Epigentek Cat# P1034), as per the manufacturer’s instructions.

### Total DNA methyltransferase (DNMT%) activity

Nuclear extracts from hamstring muscle of 35 day old WT and *Mtm1* KO mice exposed to PBS or VPA (*n* = 6/group) were obtained by the EpiQuik™ Nuclear Extraction Kit (cat. OP-0002–1; EpiGentek). Total DNA Methyltransferase (DNMT) activity was measured via calorimetric assay in duplicates using DNA Methyltransferase Activity/Inhibition ELISA Easy Kit following the manufactures protocol (cat. P-3139; EpiGentek).

### 5-Hydroxymethylation (5 hmC%) quantification

DNA was extracted from 35 day old hamstring muscle of WT and *Mtm1* KO mice exposed to PBS or VPA (*n* = 6/group) using Qiagen DNeasy Blood & Tissue Kit (cat. 69506 Qiagen). A total of 100 ng of input DNA was used to quantify 5-hydroxymethylation (5hmC%) using the MethylFlash Global DNA Hydroxymethylation (5-hmC) ELISA Easy Kit (cat. P-1032–96; EpiGentek) following the manufactures protocol. All samples were run in duplicate.

### Methionine cycle metabolites

Methionine cycle metabolites were quantified in 35 day old mouse whole skeletal muscle in collaboration with Dr. Teodoro Bottiglieri at the Center of Metabolomics at Baylor Scott & White Research Institute (Dallas, Texas, USA). Concentrations of methionine, *s*-adenosylmethionine (SAM), *s*-adenosylhomocysteine (SAH), cystathionine (Cys), choline and betaine were determined by LC–ESI–MS/MS as previously described [4]. Clear perchloric acid extracts containing labelled isotopes were injected into the LC–MS/MS system (QTrap 5500, Sciex, Framingham, MA) and metabolites quantitated using Analyst 6.0 (Sciex, Framingham, MA). The SAM:SAH ratio was calculated as an index of methylation potential.

### Reduced representation bisulfite sequencing (RRBS)

DNA was extracted from 35 day old mouse hamstring muscle using DNeasy Blood & Tissue Kit (Qiagen). RRBS was performed using the Bioconductor MethylKit package using default parameters, with the exception that overdispersion correction was performed. DMPs called based on FDR *q* value < 0.05 and methylation difference > 10%. All effect sizes for DNA methylation data are reported as “methylation difference” and calculated as the difference in mean DNA methylation between groups. A higher effect size cut-off for the RRBS analysis than the methylation array analysis was chosen because low read depth can inflate effect sizes.

### CpG methylation array

DNA was extracted from 35 day old mouse TA using phenol–chloroform extraction method. DNA samples (1100 ng) were submitted to TCAG, bisulphite converted and ran on the Infinium Mouse Methylation BeadChip. Mouse array data processed and normalized in Genome Studio using Illumina normalization and then imported into R for analysis. To identify genotype-associated CpGs, linear regression was applied to each CpG (287,050 total) using the Limma package. To identify sites at which VPA treatment corrected aberrant DNA methylation patterns in *Mtm1* KO mice, we applied an interaction model using the Limma package. We reported sites that met a nominal *p* value < 0.05, and methylation difference > 5% between PBS *Mtm1* KO vs. PBS WT (i.e. a genotype effect) and PBS *Mtm1* KO vs. VPA *Mtm1* KO (i.e. a drug effect). As well, an effect size was calculated for PBS WT vs VPA *Mtm1* KO, which was required to be < 5% and therefore, suggestive of a return to “normal” DNA methylation values following VPA treatment in the *Mtm1* KO mice.

### Patient blood samples

Genomic DNAs from peripheral blood of male patients with XLMTM were obtained in accordance with protocol 03–08-128R of the Boston Children’s Hospital Institutional Review Board, protocol 06/Q0406/33 from the MRC Centre for Neuromuscular Diseases Biobank (UK), protocol 12-N-0095 from the NIH, National Institute of Neurological Disorders and Stroke (NINDS) ethics review board, and protocol 100054920 from the Hospital for Sick Children REB. All patients in the XLMTM group from Boston Children’s Hospital were derived from a previously published cohort [7], exhibited clinical findings consistent with a diagnosis of XLMTM and were hemizygous for *MTM1* variants classified as either pathogenic or likely pathogenic under published criteria of the American College of Medical Genetics [49]. All other cases met the diagnostic criteria for XLMTM (pathogenic variant in *MTM1* and clinical features consistent with XLMTM). Total genomic DNA was extracted following standard protocols utilizing Gentra Puregene Kits (Qiagen).

### Infinium MethylationEPIC array

Human EPIC array data were generated on total genomic DNA extracted from whole blood. All samples were run in two array batches which were stratified for XLMTM cases and controls; one of these batches contained all *ACTA1* samples. We used the *minfi* package for preprocessing and normalization (Illumina normalization). Following filtering, 792,415 sites remained for analysis, including those mapping to sex chromosomes as all participants were male. Blood cell proportions were estimated using the Houseman method and the Bioconductor package “FlowSorted.Blood. EPIC”. To derive a *MTM1* signature, XLMTM patients (*n* = 19) were divided into two groups: (1) the “discovery” cohort of *n* = 12 (*n* = 7 with LOF variants, frameshift or nonsense, *n* = 1 inframe deletion, *n* = 3 splice variants, and *n* = 1 missense), and (2) the “test” cohort of *n* = 7 individuals with both LOF and missense variants, *n* = 5 and *n* = 2. The 12 discovery samples and typically developing male control individuals (*n* = 21) were used to define a set of differentially methylated positions (DMPs). First, all CpGs significantly associated with age (*n* = 24,537; FDR-corrected *p* value < 0.05) were removed. Next, a linear model was applied using LIMMA regression to all remaining CpGs with XLMTM status as the main effect and three covariates: age, estimated neutrophil proportion, estimated monocytes proportion. The remaining blood types (CD8T, CD4T, B cell, Natural Killer) were associated with neutrophil proportion and therefore not included. Sites which met a nominal significance (uncorrected *p* value < 0.005 and methylation difference 5%) were reported. These sites were then validated using PCA and hierarchical clustering in a new set of samples: the XLMTM test cohort, an additional *n* = 4 controls and *n* = 5 individuals with *ACTA1* variants. Qlucore Omics Explorer was used for data visualization, including heat maps and PCA plots. DNA methylation values in the associated figures are mean centered.

### Chromatin immunoprecipitation (ChIP)

For each (ChIP), approximately 20–30 mg of frozen tissue was cross-linked and disaggregated into single cell suspension in 1% formaldehyde diluted with Solution A (50 mM Hepes–KOH, 100 mM NaCl, 1 mM EDTA, 0.5 mM EGTA) using a Dounce homogenizer. Fixation was carried out at room temperature for 15 min and stopped with 125 mM glycine. After two washes with ice cold PBS, cells were filtered through 100 μm cell strainer. Cells were lysed using low SDS Chromatin EasyShear Kit (Diagenode) following the manufacturer’s instructions and resuspended in Shearing Buffer (Diagenode) supplemented with Protease Inhibitor Cocktail (Roche). Chromatin was shared into 200–500 bp fragments by sonication for 12 cycles (30 s ON, 30 s OFF) with Bioruptor Pico sonicator (Diagenode). An aliquot of the 10% of shared chromatin from each sample was removed for input DNA extraction. The remaining chromatin lysates were combined with 10 μg of anti-H2K27ac antibody (#39133, Active Motif) and incubated overnight rotating at 4 °C, and then with 100 μl of pre-blocked (0.5 mg/ml BSA) Dynabeads protein G beads (ThermoFisher) for 4 h. Beads were then washed six times with RIPA buffer and resuspended in ChIP Elution buffer (50 mM Tris–HCl, pH 8; 10 mM EDTA; 1% SDS). Cross-linking was reversed by overnight incubation at 65 °C. Cellular proteins and RNA were digested with Proteinase K (Invitrogen) and RNaseA (Ambion). ChIP and 10% input DNA were purified by phenol/ chloroform precipitation, and DNA concentrations were measured with Qubit 2.0 Fluorometer (Invitrogen Life Technologies).

### Chip-qPCR

Enrichment of H3K27ac in the promoter or exon regions of seven candidate genes was assessed with ChIP-qPCR using 0.25 ng of ChIP DNA and equivalent volume of the 10% input DNA for each reaction. ChIP-qPCR primers were designed in NCBI-Primer Blast [65], and confirmed for specificity of targeted PCR product by melt-curve analysis. The targets regions were predicted using the published tracks from H3K27ac/H3k4me2 in mouse quadricep muscle on the UCSC genome browser. Primers were:

*Atf3* F1: CTAGGGCTTCAGTCTCCGGT, R1: GCGAAGACTGGAGGTGAGTT;

*Bcl2l1* F: GTCTCCTTCGTCCCTTGTGG, R1: TTCCTCAGCGGATGGAAACC;

*Btg2* F1: TAAAGACACCCCAGGCAAGA, R1: CTCAAGGTTTTCAGTAGGGCG;

*Gemin5* F1: GGGTCTAGGCTGGGTCAGTC, R1: GGGAGTTCTGTAAGTCGCCG;

*Junb* F1: ATAGGGATCCGCCAGGTTGA, R1: CCGGATGTGCACGAAAATGG;

*Kcnc3* F1: ACGTGCTCAACTACTACCGC, R1: CCACGTCCGTCTCGTCTATG;

*Mettl11b* F1: GCTCTGACTCACTTACCCAGG, R1: AGATCCCGTTGGCAGAAGAC.

Each reaction was performed in triplicate using 1X advanced low-ROX qPCR master-mix with Supergreen (Wisent), 20 μM of each primer and were run using ABI Prism 7000 Real-Time detection system (Applied Biosystems). Percent input method was used for fold change calculations.

### Differential expression vs RRBS

A total of 2,424 CpGs were reported in RRBS data as significantly differentially methylated and were compared to 5,792 genes found to be differentially expressed by RNA sequencing. Using the function annotatePeak in the Bioconductor package ChIPseeker and mm10 genome assembly, the CpGs were mapped to genes using default settings (i.e., region range of TSS was ± 3000 bp from TS). Genomic regions were called as “Promoter”, “5UTR”, “3UTR”, “Exon”, “Intron”, “Downstream”, “Distal Intergenic”. Genes that mapped proximal to > 1 CpGs (identified using their Ensembl gene ID) were then overlapped with genes displaying differential expression.

### Western blot analysis

Protein was extracted from mouse quadriceps muscles or zebrafish trunks (tissue posterior to cloaca) in 1X RIPA buffer (Cell Signalling Technology) with added inhibitors. Immunoblotting for samples (30 μg) was conducted as previously described [45]. Total proteins were detected using REVERT Total Protein stain and visualized by LICOR instrument. Membranes were stripped using Restore Western Blot Stripping Buffer (Thermo Scientific). For cells, total cellular proteins were extracted with 1X Cell Lysis Buffer and samples (20 μg) were separated by SDS-PAGE and transferred on Immobilon-P membranes (Millipore). Membranes were incubated with appropriate antibodies. All protein densitometry analysis was conducted as previously described [45].

### Cell culture

All cell models were derived from commercially available C2C12 immortalized mouse myoblast lines (ATCC). For primary figures, WT and *Mtm1* KO C2C12 mouse myoblasts (ECACC 91031101) were maintained at low density in DMEM-glutamax without pyruvate (Gibco) supplemented with 20% foetal bovine serum (FBS) and differentiated in DMEM-glutamax without pyruvate supplemented with 2% horse serum (HS) at 90–95% confluency. Briefly, cells were seeded at 8,000 cells/cm^2^ on glass coverslips coated with 0.2% gelatine and treated or not with 250 μM VPA (Sigma-Aldrich). Two days after, cells were switched to differentiation medium. For supplementary figures, WT, *Mtm1* KO, and *Ryr1* KO C2C12 mouse myoblasts were seeded in 24- or 96-well plates (Falcon) either uncoated or coated with 5 μg/cm^2^ collagen, 2 μg/cm^2^ laminin, or 2 μg/cm^2^ fibronectin. Cells were grown in DMEM with 20% (v/v) FBS. At 80–90% confluency, media was changed to differentiation media (DMEM with 2% (v/v) HS 1 μg/mL insulin and 50 μg/mL gentamicin). 4 μg/mL cytosine β-D-arabinofuranoside (AraC) was used to eliminate undifferentiated myoblasts [43].

### Cell microscopy

For average area of MHC positive cells, fusion index and relative directionality calculations, cells were fixed with 3.7% formaldehyde for 10 min at room temperature, quenched with 50 mM NH_4_Cl for 10 min and permeabilized by 0.1% Triton X-100 in DPBS for 10 min. Cells were saturated for 1 h with 10% goat serum in DPBS and incubated with antibodies for 1 h at room temperature. After incubation with fluorescent secondary antibodies (Thermofisher), nuclei were stained with DAPI (Euromedex). Imaging was performed with the ZOE Fluorescent Cell Imager (Bio-Rad Laboratories). Area and fusion index were calculated using ImageJ/Fiji. Relative directionality was calculated following the protocol described in [6]. For confocal microscopy, cells were fixed with 3.7% formaldehyde, quenched with NH_4_Cl for 10 min and permeabilized with 20 μM Digitonin/PIPES-BS (PIPES 20 mM pH 6.8, NaCl 137 mM, KCl 2.7 mM) for 5 min. After a 1 h saturation period in 10% goat serum/PIPES-BS, cells were incubated with anti-Integrin β1, clone MB1.2 antibody (Sigma-Aldrich) for 2 h at room temperature. After 3 washes with PIPES-BS, Alexa Fluor 488 anti-rat secondary antibody in 10% goat serum/PIPES-BS was added for 1 h. Cells were then washed 3 times with PIPES-BS and coverslips were mounted with FluorSave reagent (Calbiochem). Imaging was performed with confocal LSM900 Zeiss microscope (Zen software, × 63 objective). β1-integrin vesicles densities were calculated with Fiji.

### Constructs for cell transfections

N174-MCS (puromycin) was a gift from Adam Karpf (Addgene plasmid # 81068; http://n2t.net/addgene:81068; RRID:Addgene_81068). V5-miniTurbo-NES_pCDNA3 was a gift from Alice Ting (Addgene plasmid # 107170; http://n2t.net/addgene:107170; RRID:Addgene_107170). N174(puro)-MTM1-miniTurbo-flag and N174(puro)-miniTurbo-flag were constructed using the following plasmids and primers using *In-fusion kit (Takara Bio)* according to the manufacturer’s instructions. For N174(puro)-MTM1-miniTurbo-flag: N174-MCS (puromycin) was linearized with BamH1.

MTM1 was amplified using the following primers:

Forward: 5′-CAGGTCGACTCTAGAGGATCCATGGACTACAAAGACGACGACG-3′.

Reverse: 5′GCAGCGGGATCGACCCACCACCTCCGGAGCCACCGCCACCGAAGTGAGTTTGCACATGGGGC-3′.

miniTurbo-flag was amplified using the following primers:

Forward: 5′-TGGTGGGTCGATCCCGCTGCTGAACGCT-3′.

Reverse: 5′-GAGCTCGGTACCCGGGGATCCTCACTTTTCGGCAGACCGCA-3′.

For N174(puro)- miniTurbo-flag: N174-MCS (puromycin) was linearized with BamH1.

miniTurbo-flag was amplified using the following primers:

Forward: 5′-CATTGTACAACGCGTGGATCCGATGATCCCGCTGCTGAACG-3′.

Reverse:5′-CTACCCGGTAGAATTGGATCCTCATTTGTCGTCGTCGTCTTTGTAGTCCTTTTCGGCAGACCGCAGAC-3′.

### Lentivirus production and transduction

Vector particles were produced as previously described [61]. C2C12 cells were transduced by incubation with lentiviral in the presence of 2 μg/mL puromycin. At 90–95% confluency, differentiations were started in the presence of 1 μg/mL puromycin.

### Identification of MTM1 interacting proteins by BioID

Proximity-dependent biotinylation (BioID) was performed according to [11]. Lysates were prepared for mass spectrometry as previously described [33].

All samples were analyzed using an Ultimate 3000 nanoRS system coupled to a Q-Exactive Plus mass spectrometer (Thermo Fisher Scientific, Bremen, Germany) operating in positive mode. Briefly, 5 μl of sample was loaded onto a C18-precolumn (300 μm inner diameter × 5 mm) at 20 μL/min in 2% ACN, 0.05% TFA. After 5 min of desalting, the precolumn was switched online with the analytical C18 nanocolumn (75 μm inner diameter × 15 cm, packed in-house) equilibrated in 95% solvent A (5% ACN, 0.2% FA) and 5% solvent B (80% ACN, 0.2% FA). Peptides were eluted using a 5–25% gradient of solvent B for 80 min, then a 25–50% of solvent B for 30 min at a flow rate of 300 nL/min. The Q-Exactive Plus was operated in data-dependent acquisition mode. Survey scans MS were acquired in the Orbitrap, on the 350–1500 m/z range, with the resolution set to a value of 70,000 at m/z 400. The 10 most intense multiply charged ions (up to 2+) were selected and fragmented by Higher Energy Collisional Dissociation (normalized collision energy set to 30%), and the resulting fragments were analyzed in the Orbitrap at 17,500 resolution. Dynamic exclusion was used within 30 s with a 10 ppm tolerance to prevent repetitive selection of the same peptide.

### Bioinformatic MS data analysis

Acquired MS and MS/MS data as raw MS files were converted to the mzDB format and processed to generate peak-lists [10]. Peak lists were searched against UniProtKB/Swiss-Prot protein database with *Mus musculus* taxonomy (96216 sequences) in Mascot search engine (version 2.6.2, Matrix Science, London, UK). Cysteine carbamidomethylation was set as a fixed modification. Methionine oxidation and acetylation of protein N-terminus were set as variable modification. Mass tolerances in MS and MS/MS were set to 10 ppm and 0.8 Da, respectively. Proline software was used for the validation and the label-free quantification of identified proteins in each sample. Mascot identification results were imported into Proline. Search results were validated with a peptide rank = 1 and at 1% FDR both at PSM level (on Adjusted e-Value criterion) and protein sets level (on Modified Mudpit score criterion). Label-free quantification was performed for all proteins identified: peptides were quantified by extraction of MS signals in the corresponding raw files, and post-processing steps were applied to filter, normalize, and compute protein intensities. The cross-assignment of MS/MS information between runs was enabled. Each protein intensity was based on the sum of unique peptide intensities and was normalized across all samples by the median intensity. Missing values were independently replaced using Perseus (Gaussian imputation). After log2-transformation of the data, the values of the technical replicates were averaged for each analyzed samples. To compare the two conditions, an unpaired two-tailed Student’s t-test was performed. Proteins were considered significantly represented when their absolute log2-transformed fold change was superior or equal to 1 and their *p* value under or equal to 0.05. Volcano plots represent log10 (*p* value) according to the log2 ratio.

### Statistics and reproducibility

Statistical analyses were performed using GraphPad Prism version 8/9. Differences were considered to be statistically significant at *p* < 0.05 (*), *p* < 0.01 (**), or *p* < 0.001 (***). All data, unless otherwise specified, are presented as Mean ± SEM. For comparisons between two experimental groups, after a normality test, data were analyzed by unpaired *t* test. A one-sample *t* test was used for comparisons with control group values that had been set to 1 for normalization purposes. For comparisons between more than two experimental groups, data were analyzed either by one-way or two-way ANOVA with a post-hoc test correcting for multiple comparisons. For each experiment, data are representative of at least three independent replications, with similar results obtained.

## Results

### Large scale drug screen in a zebrafish model of XLMTM

To facilitate drug discovery, we utilized the zebrafish *mtm1*^*zf711*^ mutant model (*mtm*) [53]. This model has an 8 bp frameshift mutation in exon 5 that exhibits a biallelic, loss-of-function phenotype. The same phenotype is observed with a second *mtm1* allele (homozygous for a 12 bp inframe deletion in the phosphatase domain; *mtm*^PD^) as well as in *mtm1* morphants (Fig. S1a). The *mtm* model displays several “screenable” abnormalities, including larval fin fold degeneration of ranging severities (Fig. 1a) [53]. We screened a library of 1280 clinical stage chemicals (US Drug Collection, Microsource) to identify suppressors of *mtm* fin degeneration (Fig. 1b, S1a–S1c). We identified 40 preliminary hits, 4 of which re-tested positive and suppressed the phenotype in pools of 250 embryos. Due to its potential for clinical translation and its unique mechanisms of action, we focused further analysis on the anti-epileptic drug sodium valproate, i.e. valproic acid (VPA).

### Valproic acid (VPA) suppresses multiple mtm phenotypes in a dose dependent manner

We first tested dose–response of VPA, and found that it increasingly suppresses the fin phenotype, with complete rescue at the highest doses (Fig. 1C). VPA also prevents fin degeneration in the *mtm*^PD^ mutant as well as in *mtm1* morphants (Fig. S1d, S1e). As fin degeneration is not strictly a skeletal muscle specific phenotype, we wanted to examine an outcome related to muscle weakness. We chose to evaluate swim behaviour, which can be quantitatively assayed using the ZebraBox platform (ViewPoint Behavior Technology). As with fin degeneration, VPA treatment improves *mtm* swimming speed in a dose-dependent manner, both with pre-symptomatic exposure and with treatment after swim defects are present (Fig. 1D, S1F). However, VPA has minimal benefit on larval survival, likely due to the fact that it does not prevent or reverse the liver abnormalities noted in *mtm1* mutants (Fig. S1G, S1H).

### *VPA modifies mtm phenotypes* via *its action as a histone deacetylase inhibitor (HDACi)*

VPA is known to have multiple potential modes of action [50], including HDAC inhibition, *myo*-inositol depletion, and sodium channel inhibition. We performed a series of chemical and genetic modifier studies to identify the mechanism(s) via which VPA suppresses the *mtm* fin phenotype. We provide several lines of evidence that it acts via its ability to inhibit histone deacetylases. First, we tested several additional chemicals that mimic specific aspects of VPA activity, and found that only HDACi could suppress the fin phenotype (Fig. 1F, S2). Importantly, valpromide, a close structural analog of VPA that lacks HDACi activity [26], does not suppress the phenotype (Fig. S2a, d). Second, morpholino mediated knockdown of *hdac1* (the single zebrafish ortholog of human HDAC1 and HDAC2) prevents *mtm* fin degeneration (Fig. 1e). Third, treatment with a histone acetyltransferase (HAT) inhibitor (anacardic acid) worsens fin abnormalities, an effect that is negated by co-treatment with VPA or another HDACi, trichostatin-A (TSA) (Fig. S2k–m). Last, we utilized chemical-chemical fingerprinting [29], an approach for determining common mechanisms of action between drugs. We built a fingerprint from 9 suppressors and 6 enhancers of the fin phenotype (see Methods), and found that VPA behaves most similarly to other HDACi (Fig. 1f).

Of note, VPA appears to modify the *mtm* fin phenotype preferentially through inhibition of class I HDACs. This was apparent because non-class I HDAC inhibitors do not suppress the phenotype (Fig. S1j–i, S2), and morpholino knockdown of *hdac3*, *hdac8*, or *hdac6* did not prevent fin degeneration (Fig. 1e). Successful HDAC target engagement was confirmed as both VPA and TSA significantly increased the acetylation state of histones H3K27 and K3K9/14 in all genotypes tested (i.e., WT, het, and *mtm* fish; Fig. 1g).

### HDAC inhibition corrects aberrant transcriptional signatures of mtm zebrafish

**Fig. 2:**
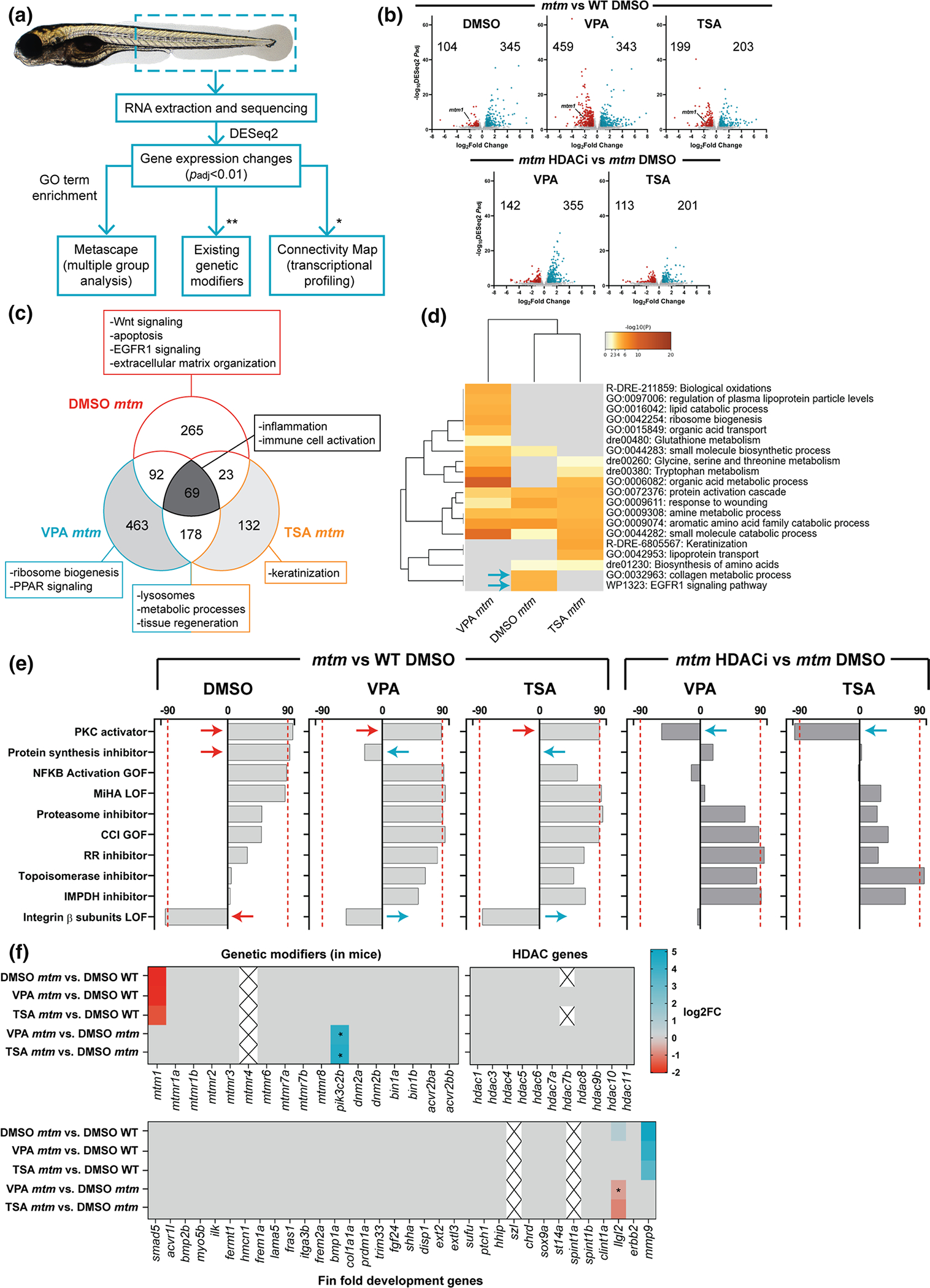
Comparative transcriptomics in *mtm* mutants treated with HDAC inhibitors. **a** Schematic outlining the analyses performed (*genes needed to be converted to human orthologs; **zebrafish orthologs of genetic modifiers identified previously in *Mtm1*^−*/y*^ mice). **b** Volcano plots showing differentially expressed genes (DEGs) between groups of interest (adjusted *p* value < 0.01, log2FC > 0.585). **c** Venn diagram highlighting DEGs that are shared or unique to each group and their associated GO enrichment terms. **d** Heat map visualization of GO terms enriched in *mtm* groups vs WT DMSO controls. Gray indicates lack of significance. **e** Connectivity scores generated by comparing the top DEGs (up to 300) from each group with the perturbagen-induced gene signature profiles in Connectivity Map. Scores > 90 indicate a high degree of similarity between queried gene lists and experimentally determined transcriptional responses to chemical or genetic perturbagens. The transcriptional signature of *mtm* mutants closely emulates that of human cell lines treated with protein kinase C (PKC) activators. **f** Heat maps showing expression changes in hypothetical modifier genes. Zebrafish orthologs of known genetic modifiers of Mtm1 in mice (*padj = 0.017, 0.015), HDAC genes, and genes involved in larval zebrafish fin fold development (*padj = 0.065) were considered. Gray indicates genes did not meet cut-offs and crossed boxes show genes that were not detected. Overall, none of these modifiers alone may account for the observed rescue

We hypothesized that the most likely means by which VPA and other HDACi abrogate the *mtm* phenotype is by modification of gene expression. To test this, we performed comparative RNA sequencing between wild type (WT) and *mtm* mutants, treated with vehicle (DMSO), VPA, or TSA (Fig. 2A). Importantly, only *mtm* fish with a mild fin phenotype were used in the DMSO group, as this enabled more accurate comparison of expression changes between groups, since severe untreated mutants completely lack fin folds. Using this approach, we identified 449, 802, and 402 differentially expressed genes (DEGs) between WT treated with DMSO vehicle and *mtm* treated with DMSO, VPA, or TSA, respectively (*p* < *0.01*, FC > 1.5) (Fig. 2b). Comparing KOs directly, there were 497 and 314 DEGs between DMSO *mtm* vs VPA *mtm* or TSA *mtm*, (Fig. 2b).

RNAseq data was subsequently evaluated using three analytic methods. First, we performed GO term enrichment, which revealed that HDACi treatment improved pathways associated with EGFR1 signalling, extracellular matrix organization, lysosomes, metabolic processes, and tissue regeneration (Fig. 2c, d, S3a). Pathways that remained dysregulated across all *mtm* groups included those related to inflammation and immune activation. Second, we used Connectivity Map, which revealed a transcriptional signature in *mtm* mutants that closely emulates one associated with protein kinase C (PKC) activation (Fig. 2e). To further explore a link with PKC signalling, we treated *mtm* fish with the pan-PKC inhibitors Ro31–8220 and Go6983, and observed no effect on the fin phenotype (Fig. S3b, c). In addition, treatment with the classical PKC activator phorbol 12-myristate 13-acetate (PMA) significantly worsened *mtm* fin degeneration, an effect that could be rescued by VPA treatment (Fig. S3d, e). Thus, while PKC activation may contribute to the fin phenotype, it does not appear to be a main driver of phenotype suppression by HDACi.

Third, we focused on expression levels of specific gene sets, including putative XLMTM modifiers, genes involved in larval fin fold development, and HDAC related genes (Fig. 2f). With the exception of *llgl2*, we observed no changes in any of these genes that could reasonably account for the HDACi effect on *mtm* fins. The observed reversal in *llgl2* expression is intriguing, as it is a cell polarity gene that regulates ErbB2 signalling and influences zebrafish fin morphology [55], but its expression alone is unlikely to explain the totality of VPA or TSA mediated rescue.

### Valproic acid and trichostatin A ameliorate aspects of the XLMTM mouse phenotype

**Fig. 3:**
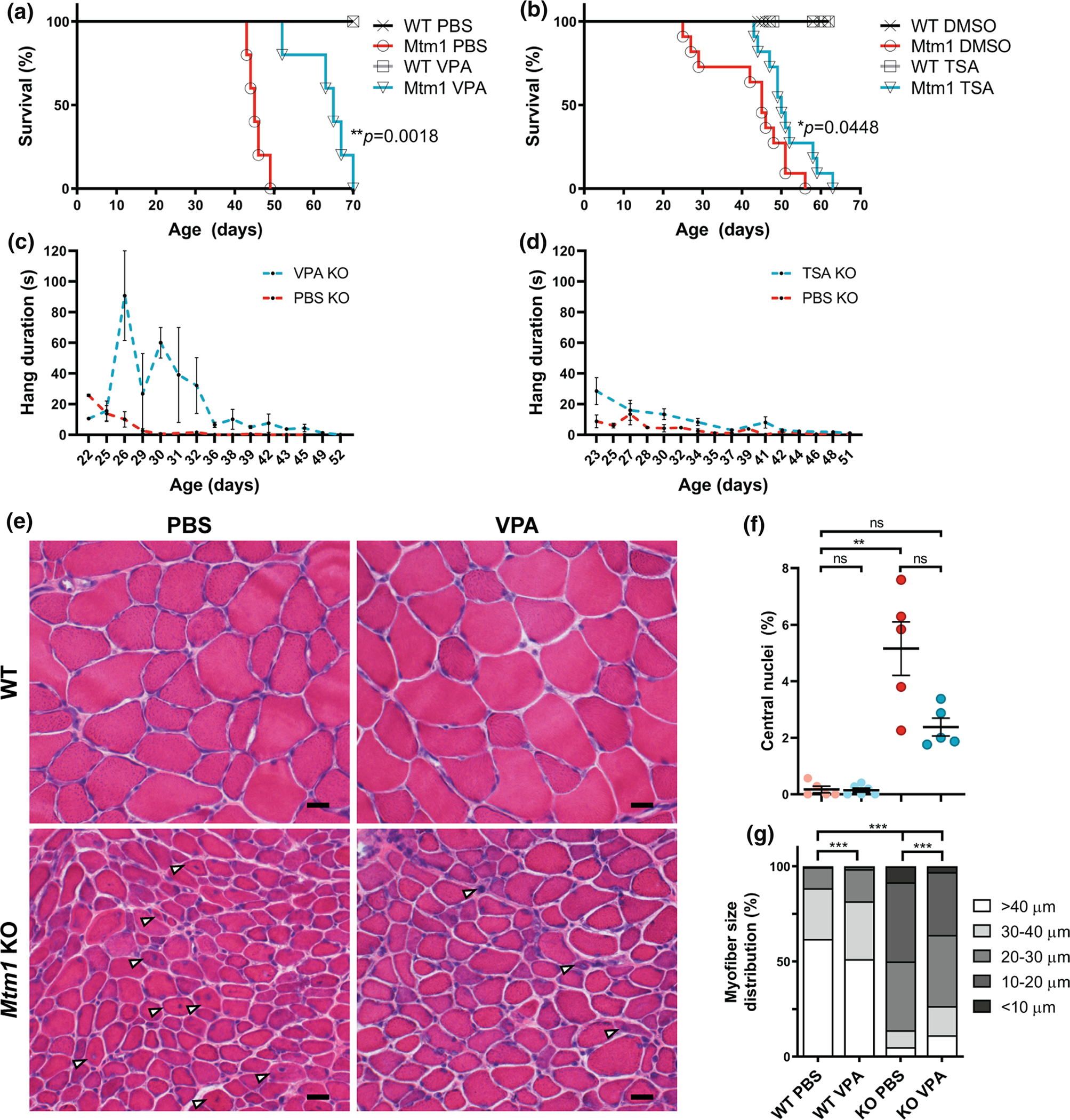
HDAC inhibitors improve phenotypic outcomes in *Mtm1* KO mice. **a**, **b** Valproic acid (VPA) treatment starting at 21 days promotes significant improvement in *Mtm1* KO survival [median survival 65 days (*n* = 5) vs. 45 days for *Mtm1* KOs + PBS (*n* = 5), **p < 0.01]. VPA improves strength as measured by wire hang duration (*n* = 2–4 per timepoint; best-fit curves by least squares regression are different, *p* = 0.0002). **c**, **d** Trichostatin A (TSA) has a measurable, yet much less positive, effect on KO mice. TSA treatment starting at 21 days promotes significant improvement in *Mtm1* KO survival [median survival 50 days (*n* = 10) vs. 45 days for *Mtm1* KOs + DMSO (*n* = 11), *p < 0.05]. TSA improves strength as measured by wire hang duration (*n* = 2–11 per timepoint; best-fit curves by least squares regression are different, *p* < 0.001). **e**, **f** Tibialis anterior muscle cross-sections at 35 days stained with haematoxylin and eosin (scale bar = 20 μm). VPA treatment is associated with a reduction in number of centrally nucleated fibres that is not significant. From left to right, Mean % central nuclei ± SEM are 0.17 ± 0.11%, 0.15 ± 0.07%, 5.20 ± 0.95%, 2.40 ± 0.32%; *n* = 1836, 2069, 6983, 6167 total fibres counted across 6–10 different fields from *n* = 5 to 6 animals; Kruskal–Wallis test with Dunn’s post-test; **p < 0.01. **g** VPA treatment significantly improves myofiber diameter size in *Mtm1* KO mice as measured by minimum Feret’s diameter. From left to right, Mean Feret’s diameter ± S.E.M are: 45.54 ± 0.388, 42.16 ± 0.332, 21.39 ± 0.185, 25.39 ± 0.224, respectively. From left to right, % myofiber size distribution for < 10 μm/10–20 μm/20–30 μm/30–40 μm/> 40 μm bins are: 0/0.497/11.019/26.678/61.806; 0.0633/1.456/16.835/30.380/51.266; 8.358/41.605/36.119/8.873/5.044; 3.033/33.037/37.323/15.326/11.282; *n* = 1207, 1580, 2716, 2473 total fibers measured across three different sections from *n* = 5–6 animals; Kruskal–Wallis test with Dunn’s post-test; ***p < 0.001

As a primary goal of this study was to identify drugs suitable for translation to XLMTM patients, we next tested valproic acid (VPA) and trichostatin A (TSA) for efficacy in an orthogonal model, the well described *Mtm1*^−/y^ knockout (KO) mouse[13]. Treatment was initiated at postnatal day 21, the time of overt symptom onset in male KO mice [14, 42, 54]. We first examined survival, which is greatly reduced in untreated KO mice (median = 37 days), and observed a significant improvement. Both VPA and TSA increased median survival by 20 and 5 days, and increased maximum survival by 21 and 7 days, respectively (Fig. 3a, c). We next tested motor function using the wire hang test. Untreated KO mice exhibit progressive decline in wire hang time. Treatment with VPA and TSA significantly improved wire hang performance, with the largest magnitude of improvement seen with VPA (Fig. 3b, d). Notably, these results show that at the doses tested, VPA provided greater benefit to *Mtm1* KO mice than TSA.

At the histological level, we examined the impact of VPA treatment on the well-defined structural changes observed in *Mtm1* KO muscle (i.e. increased central nucleation, myofiber hypotrophy, fibre type changes, and organelle disorganization) [39]. VPA reduced the number of central nuclei and increased myofiber size compared to vehicle control KOs (Fig. 3e–g, S4e). We also observed a clear improvement in fibre type composition where VPA treatment normalized the elevated ratio of type IIB to type I fibres in *Mtm1* KO (Fig. S4a, b). Conversely, we did not detect changes in appearance or number of triads (the subcellular localization of the excitation contraction coupling machinery), which are essentially absent in *Mtm1* KO mice (Fig. S4c). There was also persistence of internalized, aggregated dysferlin immunostaining (Fig. S4d), reflecting sarcolemmal disorganization. Of note, while VPA did lower the overall levels of histones H3 and H4 in skeletal muscle, it did not increase the ratio of acetylated to total protein for H3K9/14, H3K27, H4K12, or H4K16 (Fig. S5a–c, 4f–h).

### VPA and TSA correct aberrant expression signatures of *Mtm1* knockout mice

**Fig. 4:**
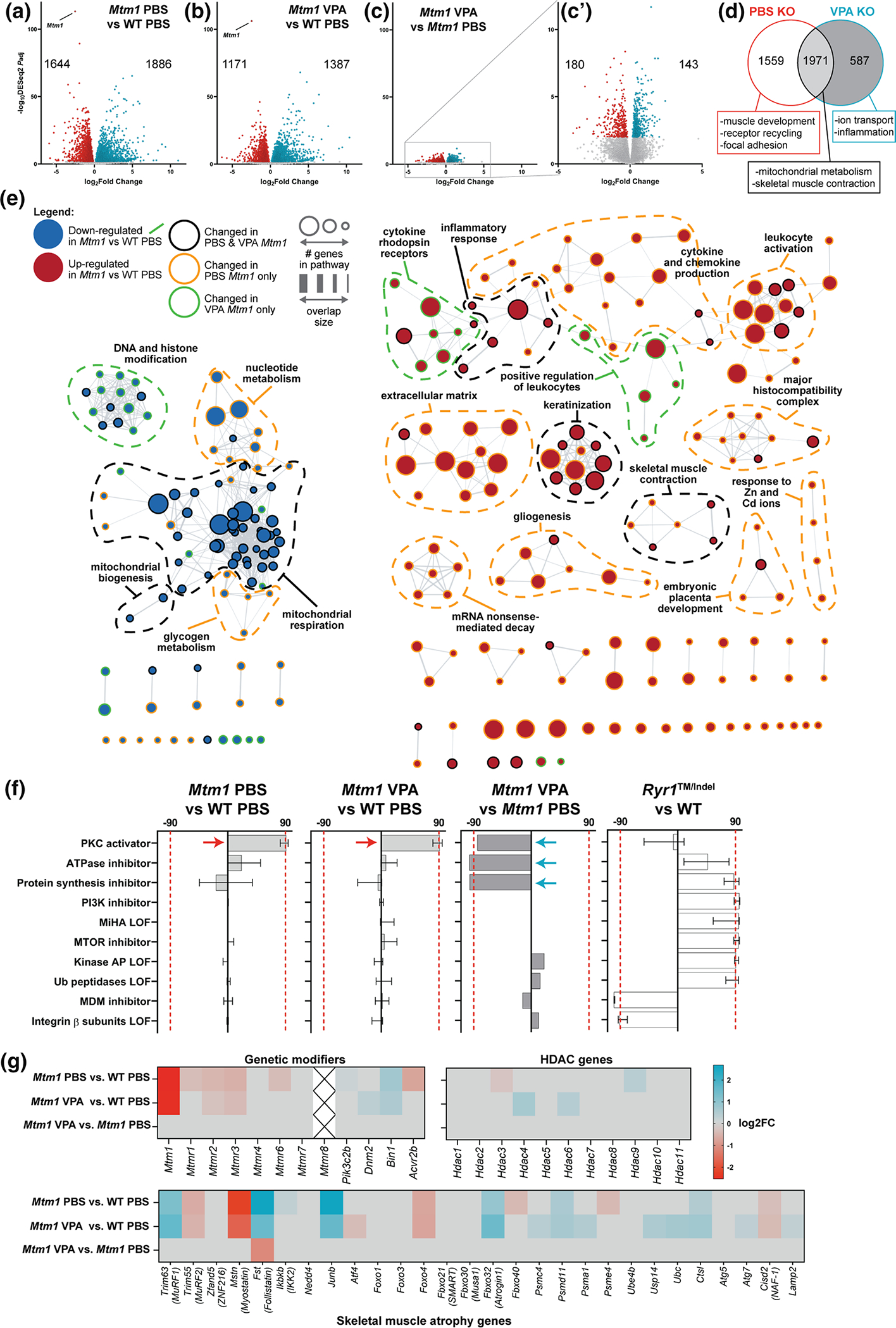
Comparative transcriptomics in *Mtm1* KO mice treated with VPA. Bulk RNA sequencing was performed on RNA extracts from tibialis anterior muscle from 35 day old mice, either treated with PBS or valproic acid (VPA) starting at 21 days. **a**–**c** Volcano plots showing differentially expressed genes (DEGs) between groups of interest (adjusted *p* value < 0.01, log2FC > 0.585). **d** Venn diagram highlighting DEGs that are shared or unique to each *Mtm1* KO treatment group compared to WT PBS and their associated GO enrichment terms. **e** Visualization of enriched GO terms in each group. Nodes specific to VPA KO are indicated with green outlines, while those specific to PBS KO are outlined in orange. **f** Connectivity scores generated by comparing the top DEGs (up to 300) from each group with the perturbagen-induced gene signature profiles in Connectivity Map. The transcriptional signature of *Mtm1* KOs closely emulates that of human cell lines treated with protein kinase C (PKC) activators. This signature is not detected in an age-matched mouse model of recessive *Ryr1*-related myopathy. **g** Heat maps showing expression changes in putative modifier genes. Gray indicates genes that did not meet cut-offs (*p*_adj_ < 0.01) and crossed boxes show genes that were not detected. The data shows that changes in the expression of modifier genes is unlikely to account for the benefit provided by VPA treatment

As with zebrafish, we hypothesized that VPA ameliorates aspects of the mouse phenotype at least partly through transcriptional modulation. We therefore performed bulk RNA sequencing, following the same analysis workflow as in zebrafish (Fig. 2a), and compared transcriptomes of 35 day old WT and KO mouse tibialis anterior muscle treated with VPA or PBS vehicle control. We identified 3,530 differentially expressed genes (DEGs) between WT PBS and KO PBS and 2,558 DEGs between WT PBS and KO VPA (*p* < 0.01, FC > 1.5) (Fig. 4a, b). KOs ± VPA shared 1971 DEGs vs WT, whereas 587 DEGs were unique to VPA KO (~ 23% of its DEGs) and 1559 DEGs (~ 44%) were unique to PBS KO, i.e., could be considered normalized by VPA treatment (Fig. 4D). Most pertinently, there were 323 DEGs between *Mtm1* KO treated with VPA and PBS (Fig. 4)C, C′). Together these data indicate partial overall transcriptional “rescue” in *Mtm1* KO mice treated with VPA.

GO term enrichment analysis revealed that VPA normalized pathway expression changes related to muscle development, receptor recycling, extracellular matrix organization, and focal adhesions, while mitochondrial metabolism and skeletal muscle contraction were not changed (Fig. 4c, S6a–c). Pathways related to DNA and histone modification genes and ERK regulation were uniquely changed by VPA, with variable effects on dysregulated inflammatory pathways. As also seen in zebrafish, transcriptional signature profiling with Connectivity Map revealed a protein kinase C (PKC) hyperactivation signature that is more pronounced in untreated KOs (Fig. 4f). Western blot analysis of PKC substrates was consistent with PKC activation in untreated *Mtm1* KOs and correction with VPA (Fig. S6d). Of note, comparison of RNAseq data to age/sex matched TA muscle from another mouse with severe myopathy, a model of recessive *RYR1* related myopathy[12], shows completely different transcriptomic signatures (Fig. 4f), supporting the specificity of PKC hyperactivation in *Mtm1* KO muscle. Lastly, we observed no difference between treated vs untreated KOs in terms of their expression of known genetic modifiers of XLMTM [21, 53], skeletal muscle atrophy associated genes [1, 9], and genes related to histone acetylation (Fig. 4g).

**Fig. 5:**
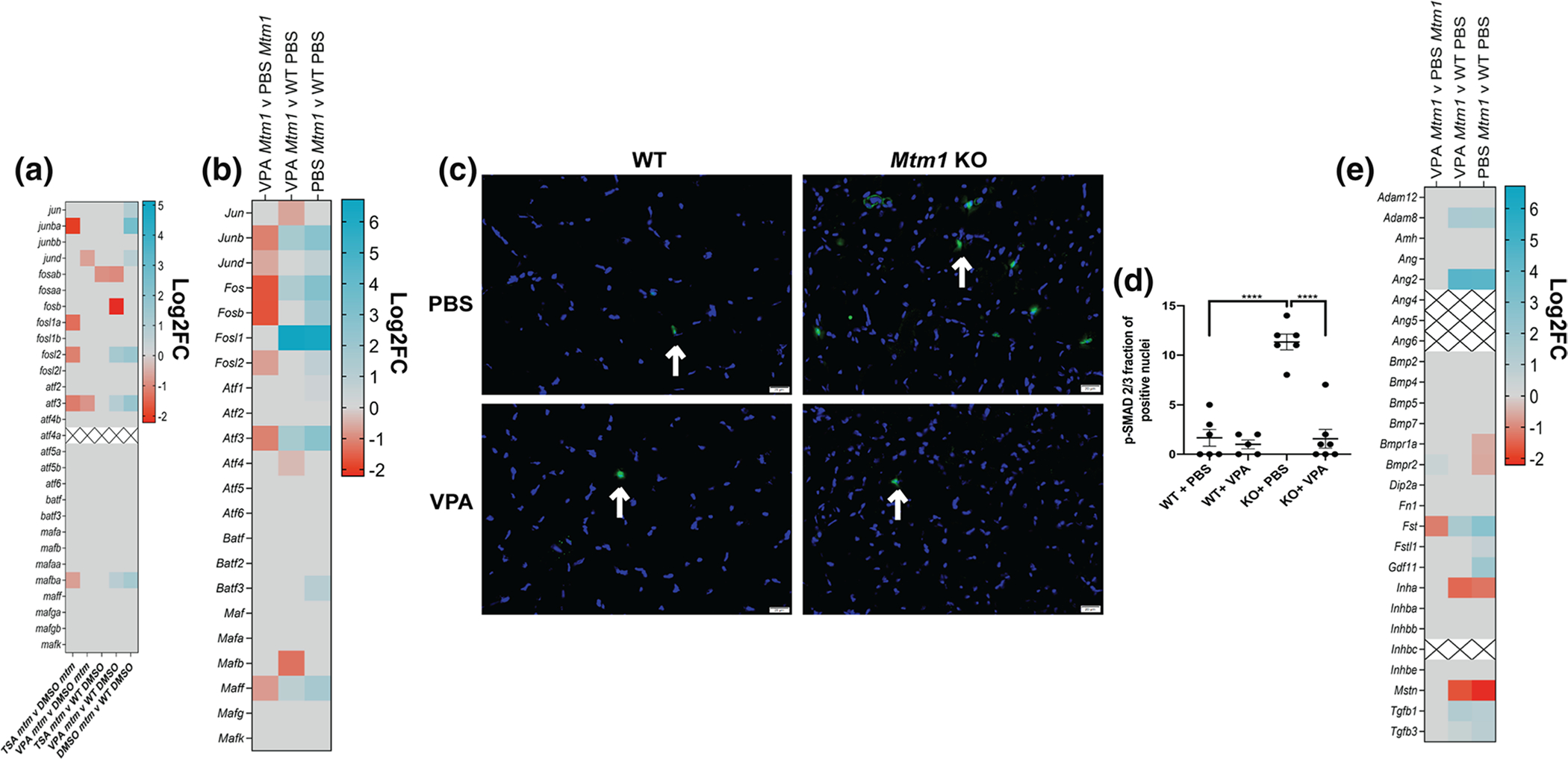
TGF beta signalling is altered in *Mtm1* KO mice and improved with VPA. **a**, **b** Heat maps showing expression changes (as determined with RNA sequencing) in AP-1 pathway genes in *mtm* zebrafish and *Mtm1* KO mice, respectively. **c** p-SMAD 2/3 immunofluorescence on tibialis anterior muscle at 35 days of age. **d** Quantification of p-SMAD 2/3 immunostaining. An increased proportion of myofibers with nuclear p-SMAD is observed in *Mtm1* KO mice. Values are Mean ± SEM. p-SMAD2/3 fraction of positive nuclei (WT + PBS &KO + PBS *n* = 6, WT + VPA *n* = 5, KO + VPA *n* = 7). PBS treated *Mtm1* = 11 ± 1 vs PBS treated WT = 1.7 ± 0.8, (****p < 0.0001) vs VPA treated *Mtm1* KOs = 1.6 ± 0.95 (****p < 0.0001) vs VPA treated WTs = 1.0 ± 0.45 (****p < 0.0001). Note that staining was confirmed to be within myofibers using double IIF with anti-dystrophin. **e** Heat map showing expression changes in follistatin interacting gene products in *Mtm1* KO mice. For all heat maps, grey indicates genes that did not meet cut-offs (*p*_adj_ < 0.01), and crossed boxes show genes that were not detected. Statistical analysis by one-way ANOVA using Dunnett’s multiple comparisons test. Scale bar = 20 μm

Similar to our zebrafish RNAseq data, we detected expression changes in AP-1 signalling pathway genes that are normalized with VPA. AP-1 pathway genes are, in part, regulated by TGF beta signalling [66], a known driver of skeletal muscle atrophy [35, 68], and thus of interest in the setting of XLMTM, where myofiber hypotrophy is a prominent feature that is rescued by VPA treatment. We thus next examined TGF beta signalling by measuring nuclear pSMAD2/3, a marker for pathway activation [32]. We found a significant increase in pSMAD positive myonuclei in KO muscle, and a significant reduction toward WT levels with VPA treatment (Fig. 5). To determine if activation of TGF beta signalling is being driven by transcriptional changes, we examined transcript levels of TGF beta pathway components. We generally did not find significant changes, with the exception of follistatin and myostatin (Fig. 5). Of note, the changes in these two genes are the opposite of what would be predicted given myofiber size in each condition and our pSMAD results [67]. They may represent compensatory changes within the myofiber, or reflect transcriptional alterations in non-muscle cell types.

### Proteomic comparison between WT and Mtm1 KO mice ± VPA identifies changes in integrins and related proteins

**Fig. 6:**
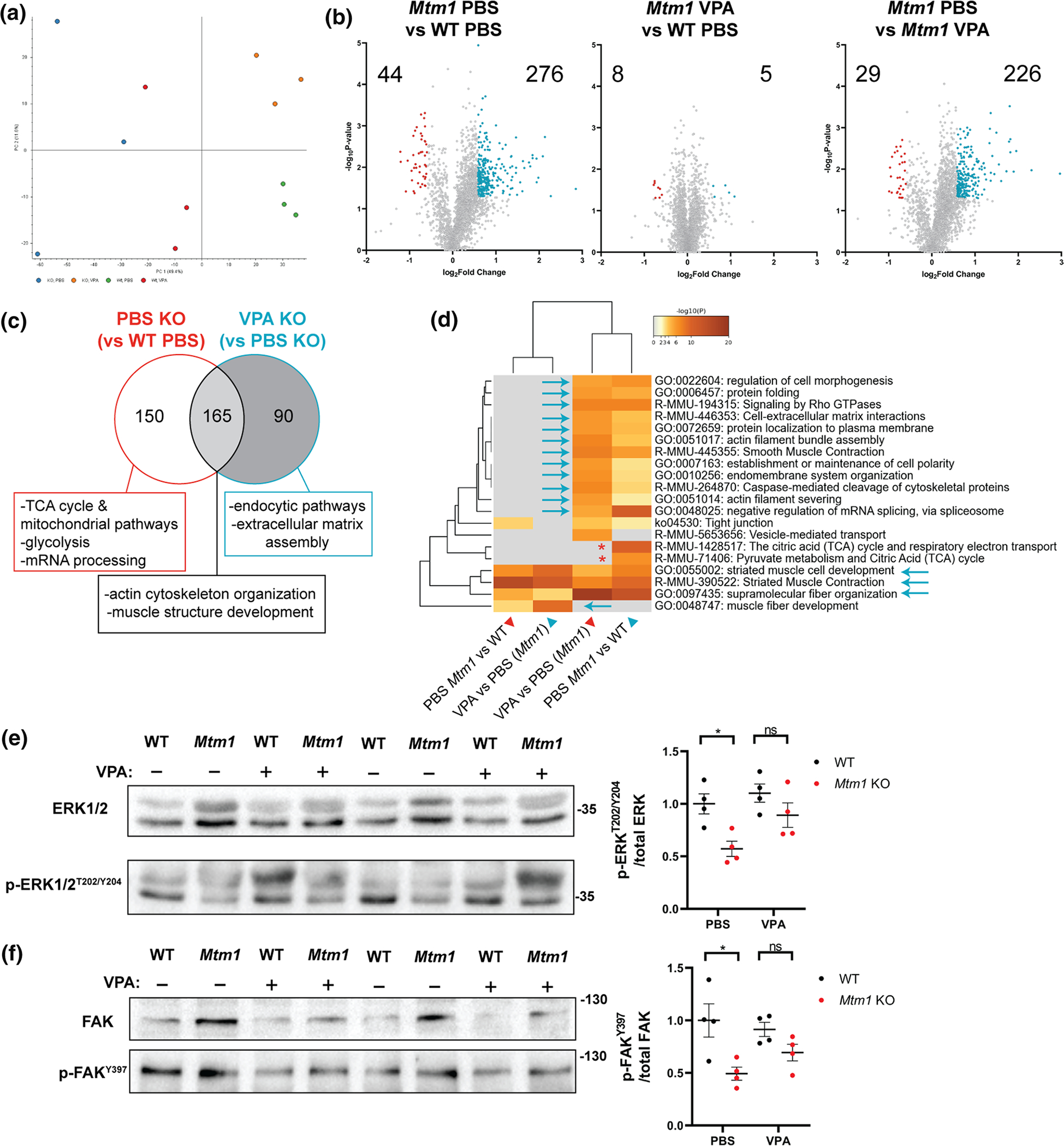
Comparative proteomics in *Mtm1* KO mice treated with VPA. Proteomes were obtained from 35 day old skeletal muscle extracts. **a** Principal component analysis (PCA) plot of each group showing that protein expression of VPA treated KO mice is more similar to WT controls than it is to PBS treated KOs. **b** Volcano plots showing differentially expressed proteins (DEPs) between groups of interest (*p* value < 0.05, log2FC > 0.585). **c** Venn diagram highlighting DEPs that are shared or unique to PBS KOs and selected associated GO enrichment terms. Given that only 13 DEPs were identified between VPA KO and WT PBS, this comparison highlights changes primarily between KOs. **d** Heat map visualization of enriched GO terms. Arrows indicate pathways that were improved by VPA treatment. **e** Western blot showing VPA treatment increases p-ERK^T202/Y204^ levels in *Mtm1* KOs (Two-way ANOVA with Tukey’s post-test; *n* = 4 each). **f** Western blot showing VPA treatment increases p-FAK^Y397^ levels in *Mtm1* KOs (Two-way ANOVA with Tukey’s post-test; *n* = 4 each)

We next performed unbiased proteomics in order to investigate protein expression changes (Fig. 6). We discovered 320 (276 up, 44 down) differentially expressed proteins (DEPs) between WT PBS and KO PBS, while only 13 (5 up, 8 down) DEPs were identified between WT PBS and KO VPA (Fig. 6b). KO VPA muscle had 255 (29 up, 226 down) DEPs compared to KO PBS. Similar to the RNAseq data, pathways related to muscle development, endocytic pathways, and extracellular matrix organization were dysregulated in KOs and normalized by VPA (Fig. 6C, Fig. S7a, b). DNM2 and BIN1 peptides (two MTM1 interactors and disease modifiers [21, 41]) were detected and were not different across samples. We validated by western blot (using separate samples) that the DNM2 elevation observed in KO muscle was not normalized with VPA (Fig. S7c), thus distinguishing VPA treatment from other current genetic [57] and pharmacological [31, 42] approaches being explored for XLMTM. Of note, VPA also does not correct elevated levels of polyubiquitinated proteins in *Mtm1* KOs (Fig. S8d).

Interestingly, our proteomic analysis revealed that several integrin-related proteins (e.g., ITGB1, TLN1, and ILK) were elevated in KOs and normalized with VPA treatment. Consistent with this, VPA reduced elevation of phospho-FAK levels in *Mtm1* KOs (Fig. 6f). We also tested whether VPA modulated ERK signalling, a pathway identified in our RNAseq data and from previous reports, and found that VPA increased phospho-ERK levels in *Mtm1* KOs (Fig. 6e).

### VPA ameliorates integrin dependent abnormalities observed in Mtm1 knockout C2C12 cells

**Fig. 7:**
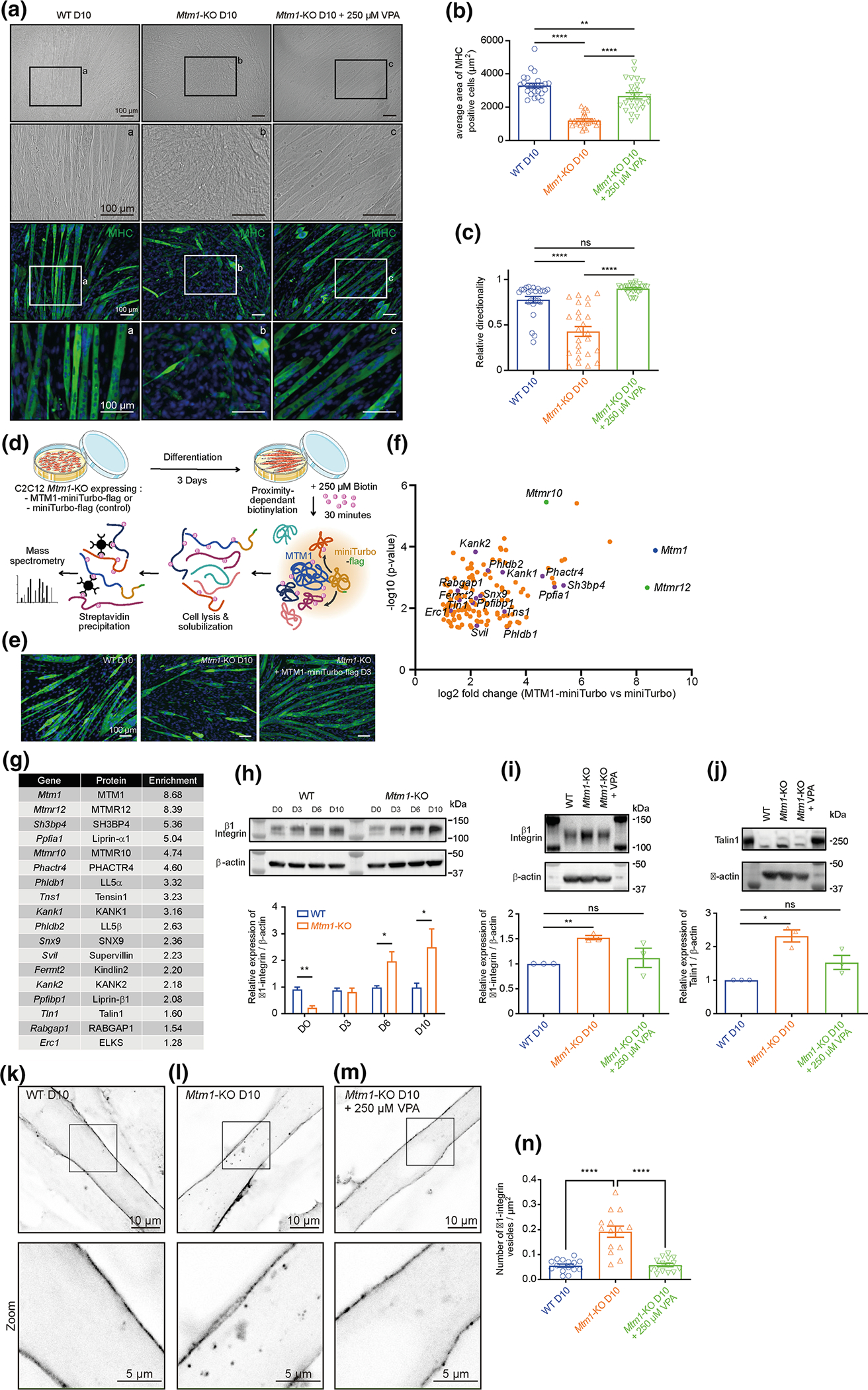
VPA rescues differentiation arrest and β1-integrin alterations in *Mtm1*-KO myotubes. **a** Brightfield of MHC and DAPI staining of differentiated C2C12 myotubes (D10) showing that VPA rescues the aberrant differentiation and reduced size observed in *Mtm1*-KO cells. Scale bars, 100 μm. **a**–**c** are higher magnifications of the indicated areas shown as boxes. **b** Average area of MHC labeled WT, *Mtm1*-KO and *Mtm1*-KO VPA-treated cells after 10 days of differentiation. Data are represented as Mean ± SEM, *n* = 5, 5 fields per independent experiment, each point represents one *field* of view, ****p < 0.0001 and **p < 0.01, one-way ANOVA test and Šídák’s multiple comparisons test. **c** Relative directionality of WT, *Mtm1*-KO and *Mtm1*-KO VPA-treated cells after 10 days of differentiation. Data are represented as Mean ± SEM, *n* = 5 fields per independent experiment, each point represents one field of view, *ns* not significant and ****p < 0.0001, one-way ANOVA test and Šídák’s multiple comparisons test. **d** BioID workflow for the identification of an MTM1 interactome. **e** MHC staining of WT, *Mtm1*-KO and *Mtm1*-KO expressing MTM1-miniTurbo-flag myotubes after the indicated days of differentiation, showing that miniTurbo does not affect MTM1 function. Scale bar, 100 μm. **f** Identification of candidate interactors by quantitative mass spectrometry. Volcano plot depicting quantified proteins (gene names). The *x* axis shows the average fold change (log_2_) in protein abundance in MTM1-miniTurbo samples in comparison to miniTurbo controls, and the *y* axis shows the −log10 (*p* value); data for both were determined by results from three independent experiments. Significantly enriched proteins in the MTM1-miniTurbo condition are displayed in orange, MTM1 bait protein displayed in blue, and known MTM1 partners in green. Proteins (gene names) related to β1-integrin trafficking in purple. **g** Enrichment (abundance ratios as log2 values (MTM1-miniTurbo—miniTurbo)) of peptides from three independent experiments (mean) related to MTM1 and β1-integrin trafficking. **h** Top: expression of β1-integrin in WT and *Mtm1*-KO C2C12 cells at the indicated differentiation time points (*n* = 3 independent experiments). Bottom: quantification of β1-integrin expression analyzed by Western. Data are represented as Mean ± SEM, *n* = 3, *p < 0.05 and **p < 0.01 according to Student’s *t* test. **i** Top: expression of β1-integrin of WT, *Mtm1*-KO, *Mtm1*-KO VPA-treated C2C12 cells after 10 days of differentiation (*n* = 3 independent experiments). Bottom: quantification of β1-integrin expression. *ns* not significant and **p < 0.01 according to Student’s *t* test. **j** Top: expression of Talin1 of WT, *Mtm1*-KO, *Mtm1*-KO VPA-treated C2C12 cells after 10 days of differentiation (*n* = 3 independent experiments). Bottom: quantification of Talin expression. *ns* not significant and **p < 0.01 according to Student’s *t* test. Top: representative confocal images of β1-integrin distribution from 10-days differentiated WT (**k**), *Mtm1*-KO (**l**) and *Mtm1*-KO C2C12 myotubes ± 250 μM VPA (**m**). Scale bar, 10 μm. Bottom: higher magnifications of the boxed area shown on the top. Scale bar, 5 μm. **n** Quantification of β1-integrin vesicles density (Mean ± SEM, *n* = 3, 5 fields per independent experiment, each point represents one field of view, ****p < 0.0001 according to one-way ANOVA test and Šídák’s multiple comparisons test

To provide additional orthogonal validation of VPA’s ability to rescue XLMTM related phenotypes, and to further dissect disease pathomechanisms and drug mechanism(s) of action, we studied a new C2C12 cell line with CRISPR/Cas9 engineered mutation in *Mtm1*. C2C12 cells are murine myoblasts that can be differentiated to form myotubes, and *Mtm1* deficient C2C12 cells display delayed differentiation and impaired cell adhesion (Fig. 7). Remarkably, treatment with VPA largely prevents these abnormalities, as we observed normal differentiation (Fig. 7a–c) and adhesion (Fig. S8) with VPA exposure.

To further explore MTM1 function and pathomechanisms, we performed proximity labelling using MTM1 fused to the biotin ligase miniTurbo [11] in *Mtm1* KO C2C12 cells. This construct rescues the *Mtm1* KO C2C12 phenotypes, indicating that the miniTurbo tag does not alter MTM1 function. The resulting BioID study identified several known and/or predicted interactors, including MTM1 itself and the phosphatase “dead” myotubularins MTMR10 and MTMR12. Of most interest, BioID also revealed several components of the focal adhesion (e.g., Kindlin2, Talin1, Sh3bp4, Kank1/2, Tensin1; Fig. 7d–g), suggesting close proximity between MTM1 and integrin-based complexes.

These BioID findings, taken together with the *Mtm1* KO C2C12 adhesion phenotype, led us to study integrin and focal adhesions in the C2C12 model. Beta1 integrin (ITGB1), as well as several focal adhesion components (such as Talin1), were mis-expressed in KO C2C12 cells and found to be internally accumulated within the KO myotubes (Fig. 7h–m), as previously observed in *mtm*-depleted Drosophila and XLMTM human muscle biopsies[48]. Remarkably, VPA treatment restored integrin levels to those of WT, and also promoted re-localization of the focal adhesion complex to the sarcolemmal membrane (Fig. 7h–m). These findings likely explain the improved cell adhesion of VPA exposed KO cells, and may account for the rescue of myotube formation. Importantly, these data are consistent with our multi-omics analyses in *Mtm1* KO muscle, which identified changes in focal adhesion components that are corrected with VPA.

### DNA methylation is altered in *Mtm1* knockout mice and corrected with VPA treatment

**Fig. 8:**
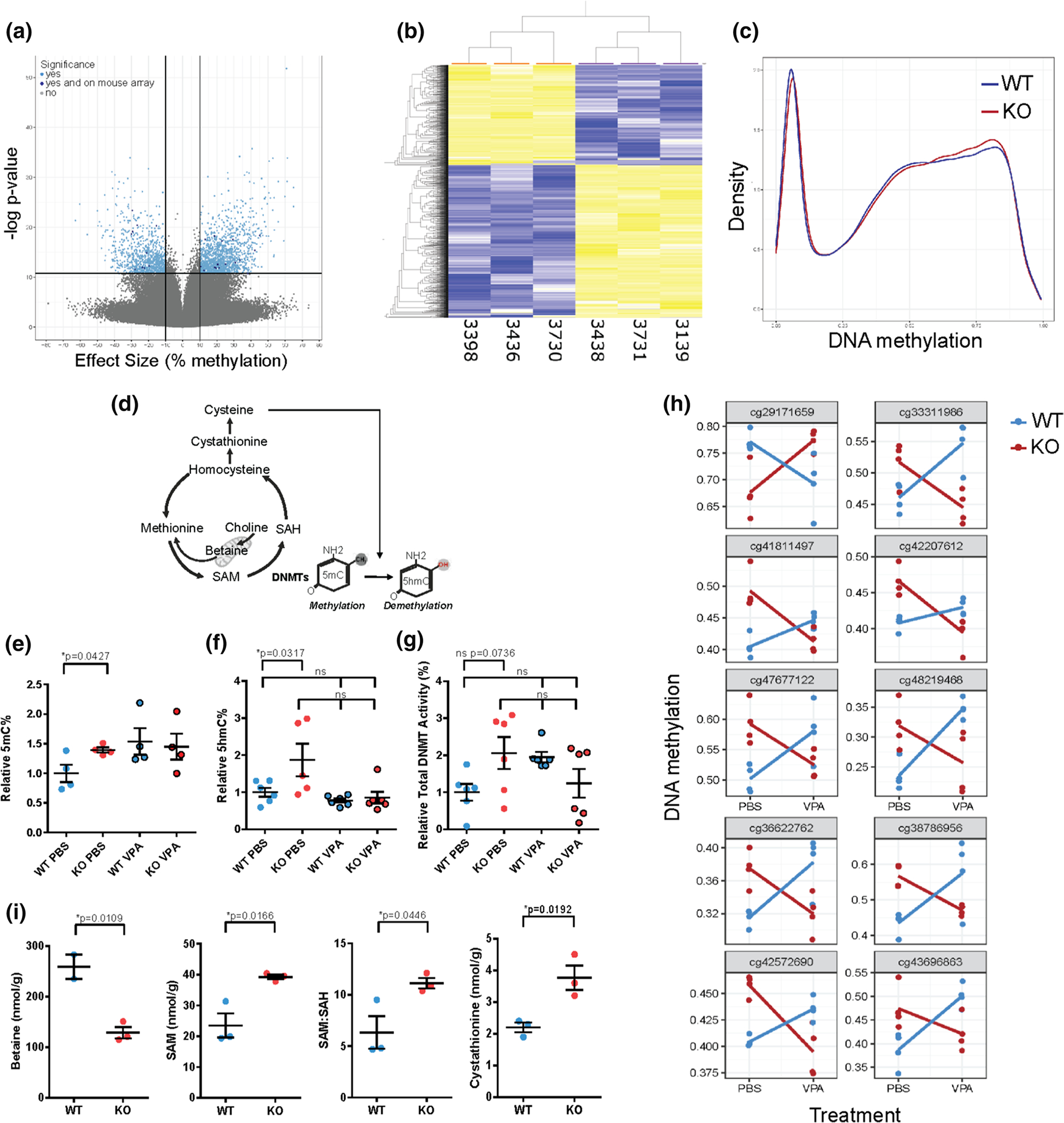
DNA methylation changes and 1-carbon metabolites in *Mtm1* KO mice. **a** Volcano plot of CpGs differentially methylated between *Mtm1* KO mice and WTs, assayed using RRBS. CpGs in light blue met a statistical threshold of 10% mean methylation difference between groups and FDR corrected **p value < 0.01. 27 CpGs in dark blue were significant on RRBS and were located within 1 kb of a significant CpGs on the mouse array. **b** Heatmap of the 2,424 CpGs identified using RRBS. Samples (*n* = 3 *Mtm1* KO mice and *n* = 3 WT) are ordered by hierarchical clustering, which shows two main clusters corresponding to genotype. **c** Density plot of all mouse array CpGs averaged across WT and *Mtm1* KO (*n* = 4 per genotype). WT distribution shows lower peak at methylated CpGs and higher peak at unmethylated CpGs, consistent with a global increase in DNA methylation in KO mice. **d** Schematic of 1-carbon metabolism and related DNA (hydroxy)methylation processes. **e** 5-methylcytosine (5mC) based ELISA shows an increase in global DNA methylation in untreated KO mouse muscle and similar levels in WT vs KO muscle treated with VPA. Values are Mean ± SEM, *n* = 4 per group. WT + PBS vs *Mtm1* KO + PBS, 3.2 ± 0.47, 4.47 ± 0.14, *p < 0.05 by Student’s *T* test. WT + VPA 4.93 ± 0.71 and KO + VPA 4.65 ± 0.7, one-way ANOVA followed by Šídák’s multiple comparisons test. Values are relative to WT + PBS controls. **f** 5-hydroxymethycytosine (5-hmC) based ELSA shows increased 5-hmC% in hamstring muscle of *Mtm1* KO compared to WT mice that is ameliorated with VPA treatment. Values are Mean ± SEM relative to WT + PBS controls. *n* = 6 per group. WT + PBS vs KO + PBS, 1.0 ± 0.12, 1.87 ± 0.44, *p < 0.05 by one-way ANOVA followed by Šídák’s multiple comparisons test. WT + VPA 0.77 ± .0.06 and KO + VPA 0.85 ± 0.16 are not different from WT + PBS controls. **g** Total DNMT enzymatic activity is not significantly increased in hamstring muscle of *Mtm1* KO + PBS compared to WT + PBS mice. Values are Mean ± SEM relative to WT + PBS controls *n* = 6 per group. WT + PBS vs KO + PBS, 1.0 ± 0.23, 2.06 ± 0.43, *p* = 0.0736 by one-way ANOVA followed by Dunnett’s post-hoc test. WT + VPA 1.95 ± .14 and KO + VPA 1.24 ± 0.39. **h** 10 CpGs with highest p-values, identified by modelling an interaction between genotype and VPA treatment (160 CpGs total nominal *p* value < 0.01 and methylation difference > 5%), in mouse array data. Most sites exhibiting hypermethylation in KOs are corrected by VPA treatment. **i** 1-carbon metabolites measured in muscle of 35 day old WT vs *Mtm1* KO mice. *Mtm1* KO mice have lower concentrations of betaine, higher concentration of SAM, and a higher SAM/SAH ratio. Values are Mean ± SEM, *n* = 2–3 per group. WT vs *Mtm1* KO, Betaine 259 ± 24, 128.8 ± 10.9, *p < 0.05; SAM 25.53 ± 3.88, 39.23 ± 0.76; SAH 3.83 ± 0.27, 3.53 ± 0.14; SAM:SAH 6.33 ± 1.59, 11.13 ± 0.50, *p < 0.05; Cystathionine 2.2 ± 0.14, 3.8 ± 0.39, *p < 0.05. Significance detected by Student’s *T* test

Our finding that HDAC inhibitors improve XLMTM preclinical phenotypes, along the known role of VPA in modulating DNA methylation [23, 44], led us to consider the role of the epigenome in XLMTM pathogenesis and VPA response. We first examined this by performing DNA methylation analysis in muscle of 35 day old WT vs *Mtm1* KO mice. Using two approaches, reduced representative bisulfite sequencing (RRBS; *n* = 3 WT, *n* = 3 *Mtm1* KO) and Infinium Methylation BeadChip array (Illumina; *n* = 4 WT, *n* = 4 *Mtm1* KO), we unexpectedly found genome-wide changes in DNA methylation in *Mtm1* KOs (Fig. 8a, b). Specifically, in hamstring muscle, we detected 2,424 differentially methylated positions (DMPs, *q* < 0.01, methylation difference > 10%) via RRBS, the majority (60.5%) of which were hypermethylated in *Mtm1* KOs. Notably, these DMPs were distributed across chromosomes and mapped to both inter- and intra-genic regions (Fig. S9).

We also quantified global DNA methylation using RRBS and methylation array and found in each data set a significant gain of methylation in the *Mtm1* KO mice as compared to WT (Wilcoxon rank sum *p* values < 2.2 × 10^−16^; Fig. 8c). Since each platform carries biases related to which CpGs are assayed, we validated this observation with a global 5-methylcytosine (5mC) based ELISA (Fig. 8d), and again found a global increase in DNA methylation in KO compared to WT mice. Next, we considered the impact of VPA on DNA methylation changes. While VPA treatment did not reduce global DNA methylation levels in KOs as compared to untreated mice (Fig. 8E), methylation levels were similar between VPA exposed WT and KO muscle, and we identified restoration of several gene-specific methylation changes by VPA (Fig. 8h). Using the methylation array, we identified 160 candidate DMPs at which altered DNA methylation levels in KO mice were ameliorated to WT-like levels after being treated with VPA (nominal *p* value < 0.01 and methylation difference > 5%; 10 DMPs with lowest p-values illustrated in Fig. 8h).

VPA may modulate the DNA methylome by affecting the activity of DNA methyltransferases (DNMTs) that carry out the transfer of active methyl groups, or by regulating demethylation processes. As such, we next measured total nuclear DNMT activity and 5-hydroxmethylation via ELISA in mouse hamstring muscle. While DNMT activity was not significantly modulated by VPA treatment (Fig. 8f), we observed global changes in 5-hydroxmethylcytosines (5hmC%), with KOs having higher 5hmC% compared to WT mice that was restored to WT-like levels by VPA treatment (Fig. 8g). Our findings are in line with a known function of VPA in affecting the epigenome through tissue specific hydroxymethylation processes, and thus suggests VPA may dynamically regulate the balance in skeletal muscle between methylation and demethylation processes [24, 64].

### Abnormal one-carbon metabolism as one explanation of epigenetic alterations in XLMTM

Perturbations in 1-carbon metabolism are one driver of epigenetic alterations. Accordingly, we measured 1-carbon metabolites in WT vs KO muscle (Fig. 8i) and found multiple abnormalities. Specifically, we observed > 1.5-fold increases in the methyl donor S-adenosyl methionine (SAM) and in the ratio of SAM:*S*-adenyl homocysteine (SAH), plus a corresponding decrease in the alternative methyl donor betaine. This is consistent with 1-carbon metabolic changes that would promote DNA hypermethylation in KO mice. We also found a ~ 1.7-fold increase in levels of cystathionine, an intermediary metabolite in the trans-sulfuration cycle, supporting the global increases in 5hmC% observed [47].

### Transcriptional changes and DNA methylation are interrelated in *Mtm1* KO mice

**Fig. 9:**
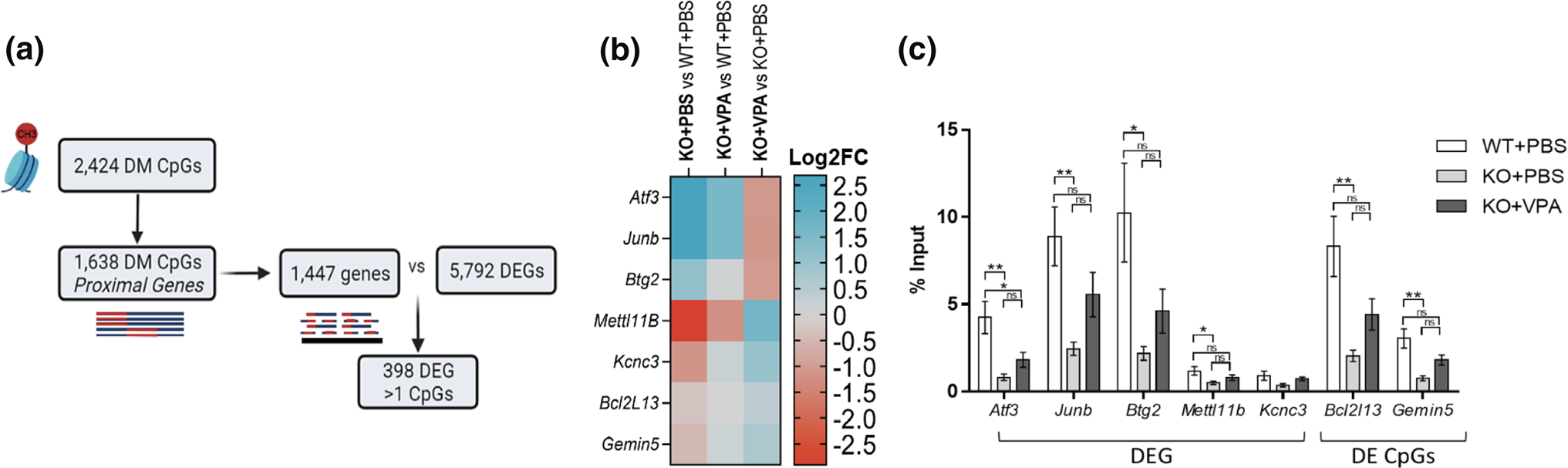
Epigenetic signatures associate with transcriptomic changes in *Mtm1* KO mice. **a** Workflow analysis to identify the correlation between differentially methylated (DM) CpGs identified by RRBS with total differentially expressed genes (DEGs) identified by RNAseq in *Mtm1* KO mouse muscle. Of the 2434 DM CpGs, 1638 mapped to various features of proximal genes, encompassing 1447 known gene targets. Within these 1447 genes, a total of 398 had significantly altered expression. **b** Heat map showing Log2FC of target genes selected for ChIP-qPCR experiment. All genes were DEG in KO + PBS vs WT + PBS mice (FDR **padj < 0.01) and their expression was modulated by VPA treatment. Blue indicates increased expression; red indicates decreased expression between comparisons. **d** ChIP-qPCR data on selected DEGs or genes with DM CpGs calculated as % input of DNA. WT mice exhibit widespread increases in H3k27ac enrichment compared to KO + PBS that is modulated by VPA. *n* = 6 biological replicate per group with *n* = 3 technical replicates per qPCR experiment. Values are expressed as Mean ± SEM. Analyses performed using one-way ANOVA at *p < 0.05

We sought to understand whether the transcriptomic changes identified in *Mtm1* KO mice were correlated with variations in DNA methylation, and to define the functional epigenetic consequences induced by VPA at these loci. Of the 2424 differentially methylated CpGs reported in our RRBS data set, 1638 CpGs mapped to various features in proximal (i.e. potentially regulatory) regions of 1447 unique genes. Within these unique genes, 389 were found to be DEG by RNAseq and proximal to > 1 differentially methylated CpG (Fig. 9a), suggesting that the transcriptomic changes may be a consequence of altered DNA methylation. In line with the known role of VPA as an HDACi, we next performed ChIP-qPCR to examine enrichment of H3K27ac at or near the promoters of selected gene loci (Fig. 9b, c). We observed an overall reduction in H3K27ac enrichment at these sites in KO mice as compared to WT, changes that are restored with VPA treatment. These findings suggest that VPA treatment has the potential to de-repress methylation signatures and modulate transcription to ameliorate aspects of the disease phenotype.

### XLMTM patients have an abnormal, unique DNA methylation signature

**Fig. 10:**
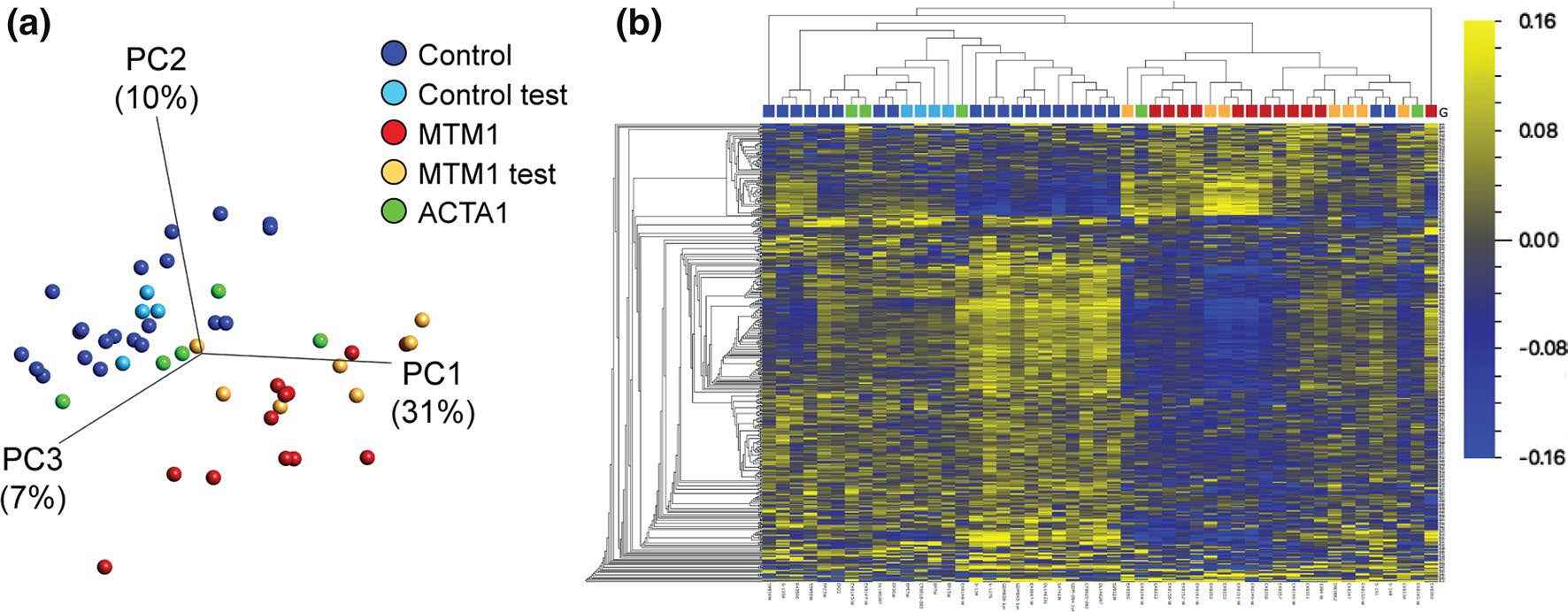
DNA methylation changes in XLMTM patients. **a** Principal component plot of 416 CpG sites identified as differentially methylated in XLMTM (data generated on Illumina EPIC array platform). *n* = 12 discovery samples were used to identify signature (plotted in red), with *n* = 21 discovery “control” males (dark blue). Test samples used to validate the differential methylation signature include *n* = 7 additional XLMTM cases (MTM1 test; orange), *n* = 4 additional controls (Control test; light blue) and *n* = 5 ACTA1 samples (green). Axes represent the first three principal components (PCs) and the total variability for which they account. **b** Heatmap and hierarchical clustering of the CpG sites and samples plotted in **a**. Each column represents a patient sample (coloured as in **a**) and each row represents a single CpG (both ordered by Euclidean clustering). Heatmap colors represent scaled and mean centered percent methylation

To determine if the novel epigenomic alterations identified in *Mtm1* KO mice are relevant to human XLMTM, we applied to XLMTM patient samples an established analytical pipeline for characterizing DNA methylation changes in rare genetic disorders of epigenetic regulation [16, 19, 51]. We examined genome-wide DNA methylation on blood samples using the Infinium MethylationEPIC array from male XLMTM patients (*n* = 19) and typically developing male control individuals (*n* = 25). XLMTM patients were divided into two groups: 1) a “discovery” cohort of *n* = 12 individuals, and 2) a “test” cohort of *n* = 7 individuals (Fig. 10a, b). Similarly, *n* = 21 age-matched controls were included in the discovery group and the remaining *n* = 4 were used in the test group. Discovery cases and controls were examined using linear regression, which revealed widespread methylation differences between control and XLMTM samples. Specifically, 416 DMPs were identified (nominal *p* value < 0.005 and methylation difference > 5%). Together these DMPs represent an abnormal XLMTM DNA methylation pattern capable of distinctly clustering all discovery XLMTM cases from controls using both hierarchical clustering and principal component analysis (Fig. 10a). To further evaluate this novel DNA methylation pattern, we tested the DMPs against our test set of XLMTM samples (*n* = 2 with missense variants and n = 5 with a LOF variants, i.e. nonsense or frameshift), plus *n* = 5 patients with another form of severe myopathy (ACTA1-related nemaline myopathy) (Fig. 10a, b). The majority of samples (except 1 XLMTM and 1 ACTA1 case) correctly clustered, thus validating the novel XLMTM-specific DNA methylation pattern and confirming epigenetic alteration as a disease feature present in patients and pre-clinical models.

## Discussion

XLMTM is a severe disease without approved treatment and with still incomplete understanding of disease pathogenesis. To address these barriers, we performed a large scale “repurposing” drug screen in a zebrafish model of XLMTM, followed by comprehensive evaluation in the fish and orthogonal studies in mouse and cell models of the disease. This experimentation led to the discovery of a new disease pathomechanism, i.e., alteration of the epigenome, critical to the XLMTM disease process. It also resulted in identification of a new therapeutic strategy for XLMTM, e.g. epigenetic modulation via HDAC inhibition, and specifically identified valproic acid as a promising drug treatment suitable for future potential translation to XLMTM patients. In total, therefore, these data advance the understanding of XLMTM and impact its therapeutic potential.

The first main finding is that valproic acid, and to a lesser extent trichostatin A, can significantly ameliorate disease relevant phenotypes in zebrafish, mouse, and cell models of XLMTM. VPA is a drug in common use in humans, used primarily as an anti-seizure therapy in children and adults with treatment refractory epilepsy. This makes it an attractive candidate therapeutic for translation to XLMTM patients. There are no reported data on VPA exposure in XLMTM patients, as epilepsy is not a primary feature of the condition. Additional future steps to translation should include dose finding and pharmacodynamic studies in *Mtm1* KO mice, as well as more in-depth assessments of safety in the pre-clinical model paradigm. To that end, there is a theoretical safety concern related to VPA, given its associated hepatoxicity and the emerging recognition of liver phenotypes in XLMTM patients [46]. No adverse events were noted in VPA treated *Mtm1* KO mice, though VPA did not appear to alter the liver phenotype noted in *mtm* zebrafish.

VPA is a drug with multiple known mechanisms of action. Several lines of evidence in our study suggest that HDAC inhibition is the primary means via which it ameliorates pre-clinical disease phenotypes. Particularly compelling is that trichostatin A, a *pan*-isoform HDAC inhibitor, also rescues elements of the disease in both zebrafish and mice. Correspondingly, treatment with VPA reversed changes in gene-specific DNA methylation and hydroxymethylation as well “corrected” disease relevant gene expression changes. Specific altered pathways of most interest to disease phenotypes include muscle development, integrin signalling, AP-1 signalling, and immune response. Importantly, elucidation of this mechanism led directly to our identification of epigenomic alternations as a novel, important element of XLMTM pathogenesis.

It is probable, however, that VPA acts upon pathways in addition to histone acetylation and DNA methylation. This is supported by the fact that VPA is more efficacious in *Mtm1* KO mice than TSA, though this difference could also be related to drug bioavailability in mice and the extent to which each drug penetrates skeletal muscle. It is also suggested by the findings in C2C12 cells, where VPA treatment ameliorated abnormalities in integrin subcellular localization, and promoted improvements in integrin dependent phenotypes (such as cell attachment). This direct impact on integrin signalling is more likely to be related to integrin trafficking, a process known to be regulated by MTM1 and to be abnormal with MTM1 deficiency [48]. The exact means via which VPA improves integrin localization and rescues integrin dependent phenotypes is not clear. A sensitization screen in yeast identified membrane trafficking as a key pathway modulated by VPA [45]; however, it did not provide insight into how it worked in this context. Future study will be required to parse out this intriguing aspect of the VPA/MTM1 interplay.

The second major finding is that epigenetic alterations appear to be a primary and important aspect of XLMTM disease pathogenesis. This is highlighted particularly by the finding of widespread and unique changes in DNA methylation in both *Mtm1* knockout mice and in XLMTM patients. We hypothesize that these changes are driven, at least in part, by functional impairment of the mitochondria in relation to their role as modulators of one carbon metabolism and control of methyl donor content for DNA (hydroxy)methylation. This is supported by our findings of increased SAM and SAM:SAH ratio, our comparative transcriptomic data, and the pre-existing knowledge of mitochondrial dysfunction in XLMTM [54], which may underlie perturbations in 1-carbon metabolism. In addition, there are multiple potential pathways that can influence the epigenome and that are abnormal in XLMTM. These include TGF beta signalling, altered intracellular calcium homeostasis, and changes in the PI3K/AKT pathway. Future experimentation will be required to dissect the potential contributions of these cellular events on epigenetic alternations in XLMTM, and the interplay between these pathways and VPA treatment.

There may also be a more direct role for MTM1 in regulating the epigenome. MTM1 contains a SET interacting domain, and SET proteins as a class typically have DNA methyltransferase activity. One early study of MTM1 function suggested expression of at least some MTM1 in the nucleus [22], though most subsequent evaluations of MTM1 subcellular localization have not corroborated this. We attempted to identify SET proteins that may interact with MTM1 by using BioID based proximity labelling, but did not identify any candidates, nor did we find other interacting factors which might directly influence the epigenome. Using IP-MS and a HIS tagged “knock in” *Mtm1* mouse model, we did uncover association between MTM1 and several nuclear proteins (MTMR12, RTN2, and NR4A3), suggesting the possibility that MTM1 may act in the nucleus as well as the sarcoplasm [54]. Of note, MTM1 is responsible for producing the majority of PtdIns5P [59], and PtdIns5P has been implicated as a regulator in skeletal myoblasts of the epigenome and of basal transcription [56]; altered PtdIns5P metabolism may thus be another avenue via which MTM1 loss influences the epigenome.

The exciting observation of a unique DNA methylation signature in whole blood from XLMTM patients lends support to the translatability of the findings in this study, and might provide a readily measurable biomarker with which to evaluate the effectiveness of novel therapies. While future work is necessary to validate our findings, such definitive DNA methylation patterns are commonly only identified in cancers and disorders caused by pathogenic variants in gene encoding epigenetic regulators [18, 19, 51]. It is also intriguing to consider in terms of the clinical phenotype of XLMTM. Unlike most other congenital muscle diseases, patients with XLMTM are often large (i.e., growth parameters > 95%), have long, unusually appearing digits, and have a unique dysmorphic facial gestalt [40]. These features are reminiscent of primary disorders of the epigenome, which frequently are associated with alterations in body size and with dysmorphic features [15, 27]. It is therefore possible that these previously unexplained features of XLMTM may, in fact, be the result of the developmental impact of primary epigenetic changes. In terms of the skeletal muscle, we suggest that the main impact of altered epigenetic programming is on myofiber size, as there is a known role for the epigenome in controlling the establishment of myofiber size [52], and treatment with VPA significantly improves this in *Mtm1* knockout muscle. Of note, a previous study of muscle biopsies from XLMTM patients identified alterations in the RNA and protein levels of several regulators of the epigenome [5], again supporting the relevance of this pathway to XLMTM pathogenesis.

In conclusion, we have identified epigenetic alterations as a novel pathomechanism in XLMTM, and found that modification of the epigenome via HDAC inhibition is a promising therapeutic strategy for this fatal disorder. MTM1 is the canonical member of the myotubularin family, and pathogenic variants in other myotubularins are associated with a range of neurogenetic disorders that currently have no treatment. In addition, XLMTM is one subtype of a family of neuromuscular disorders, called centronuclear myopathies, that share common pathomechanisms. It is therefore intriguing to consider epigenetic alterations and response to HDAC inhibition as a shared feature these MTM1 related disorders. Examination of this possibility will form the basis of future investigation.

## Supplementary Material

Supplement

## Data Availability

All data needed to evaluate the conclusions in the paper are present in the paper and/or the Supplementary Materials. RNAseq and proteomics data have been reposited to the relevant databases (i.e. GEO for RNAseq, MassIVE and ProteomeXchange for proteomics).

## Abbreviations

XLMTM: X-linked myotubular myopathy
DNM2: Dynamin-2
HDACi: Histone deacetylase inhibitor
VPA: Valproic acid
TSA: Trichostatin A
DEGs: Differentially expressed genes
DEPs: Differentially expressed proteins
DMPs: Differentially methylated positions
