# Supplementary material for: X-linked myotubular myopathy is associated with epigenetic alterations and is ameliorated by HDAC inhibition
Source: Acta Neuropathol. Author manuscript; Available in PMC 2022 Sep 1. (PMC9381459; doi:10.1007/s00401-022-02468-7)
Supplement: Supplement [file NIHMS1827549-supplement-Supplement.pdf]

Supplementary Figures

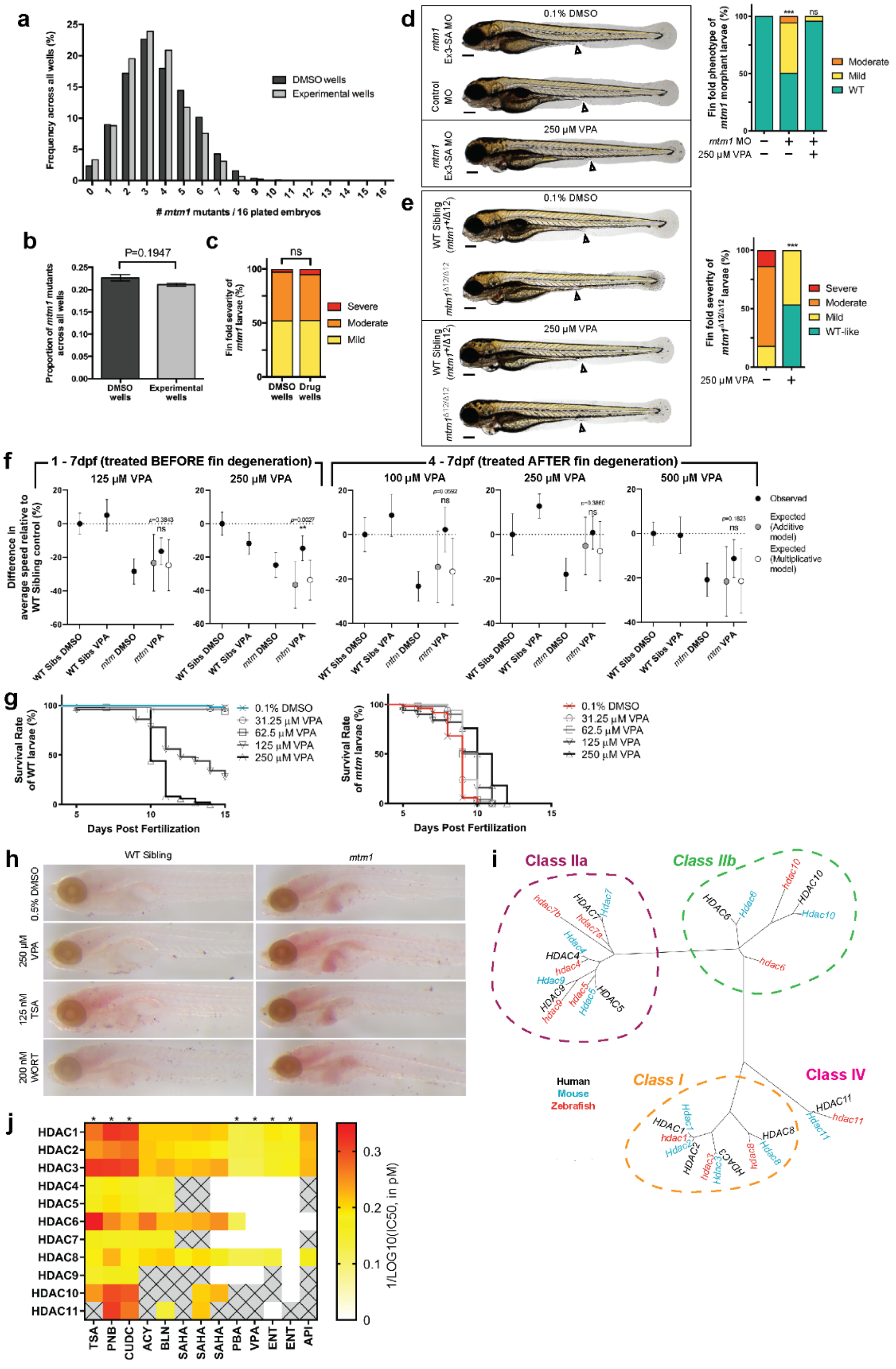

Supplementary Fig. 1. Phenotypic screen with *mtm* mutants identifies sodium valproate (valproic acid; VPA) as a suppressor of fin degeneration. A: The majority of wells contained 3-4 mutants per 16 embryos that were plated. DMSO wells (n=256) had an average of 3.625 ± 0.113 mutants per 16 embryos

and experimental wells (n=1191) had 3.377 ± 0.049. As shown, the number of experimental wells with 0 mutants was greater than in DMSO wells, suggesting some of these represented true phenotypic suppression. **B:** The average proportion of *mtm1* mutants randomly distributed into wells was 0.223 ± 0.007 for DMSO wells and 0.211 ± 0.003 for experimental wells which was not statistically different (Kolmogorov-Smirnov non-parametric test to compare cumulative distributions). Proportion values indicate Mean ± S.E.M. **C:** Fin fold severity was no different between DMSO wells and drug wells. Altogether, these data show that the screen performed as expected and it was reasonable to re-test experimental wells with 0 mutants. **D:** VPA suppresses fin degeneration in an in-frame *mtm1* mutant that is predictive to inactivate the catalytic phosphatase domain. **E:** VPA suppresses the *mtm1* morphant fin phenotype. **F:** Motor activity was consistently higher for *mtm* mutants with various VPA exposure protocols indicative of a positive chemical-genetic interaction (2-way ANOVA with Tukey's post-test; n=22-24 larvae each group) **G:** VPA does not appreciably improve survival of *mtm* mutants when treatment begins at 1 dpf (n=50 each). The median survival of mutants was 9, 9, 9.5, 10, and 10.5 days for DMSO, 31.25 μM VPA, 62.5 μM VPA, 125 μM VPA and 250 μM VPA, respectively. The median survival of WT siblings was 12 days and 10 days for 125 μM VPA and 250 μM VPA, respectively, and larvae did not grow beyond the size of ~7 dpf larvae. WT siblings exposed to 62.5 μM were smaller in size compared to the DMSO counterparts beginning at 8 dpf, suggesting developmental toxicity occurred at this concentration. In addition, 125 μM and 250 μM VPA caused pericardial and yolk sac edema in both WT and *mtm1* larvae. **H:** Neither VPA, trichostatin A (TSA), nor PI3K inhibitor wortmannin (WORT) prevented development of fatty livers at 7 dpf in *mtm* mutants as shown by Oil Red O staining. This could partially explain why VPA does not improve *mtm* survival. Larvae were exposed to chemicals beginning at 4 dpf. **I:** Maximum likelihood phylogenetic tree of 33 full-length histone deacetylase (HDAC) proteins from human, mouse, and zebrafish. **J:** Heat map illustrating the selectivity of 10 HDAC inhibitors on purified human/recombinant HDACs and their activity against the *mtm* fin phenotype. Hatched boxes indicate IC50 values were not determined for the given pair. Each column represents findings from an individual study [VPA/PBA [27]; TSA/SAHA/ENT [39]; CUDC/SAHA/PNB [49]; API/SAHA [37]; BLN/ENT [30]; ACY [56]]. Inhibition of class I HDACs is common to the six HDACi's that suppress the fin phenotype (indicated by asterisks). Abbreviations: TSA=trichostatin A; PNB=Panobinostat; CUDC=CUDC-907; ACY=ACY-1215/ricolinostat; BLN=belinostat; SAHA= suberanilohydroxamic acid/vorinostat; PBA=4-phenylbutyrate; VPA=valproic acid; ENT=entinostat/MS-275; API=apicidin.

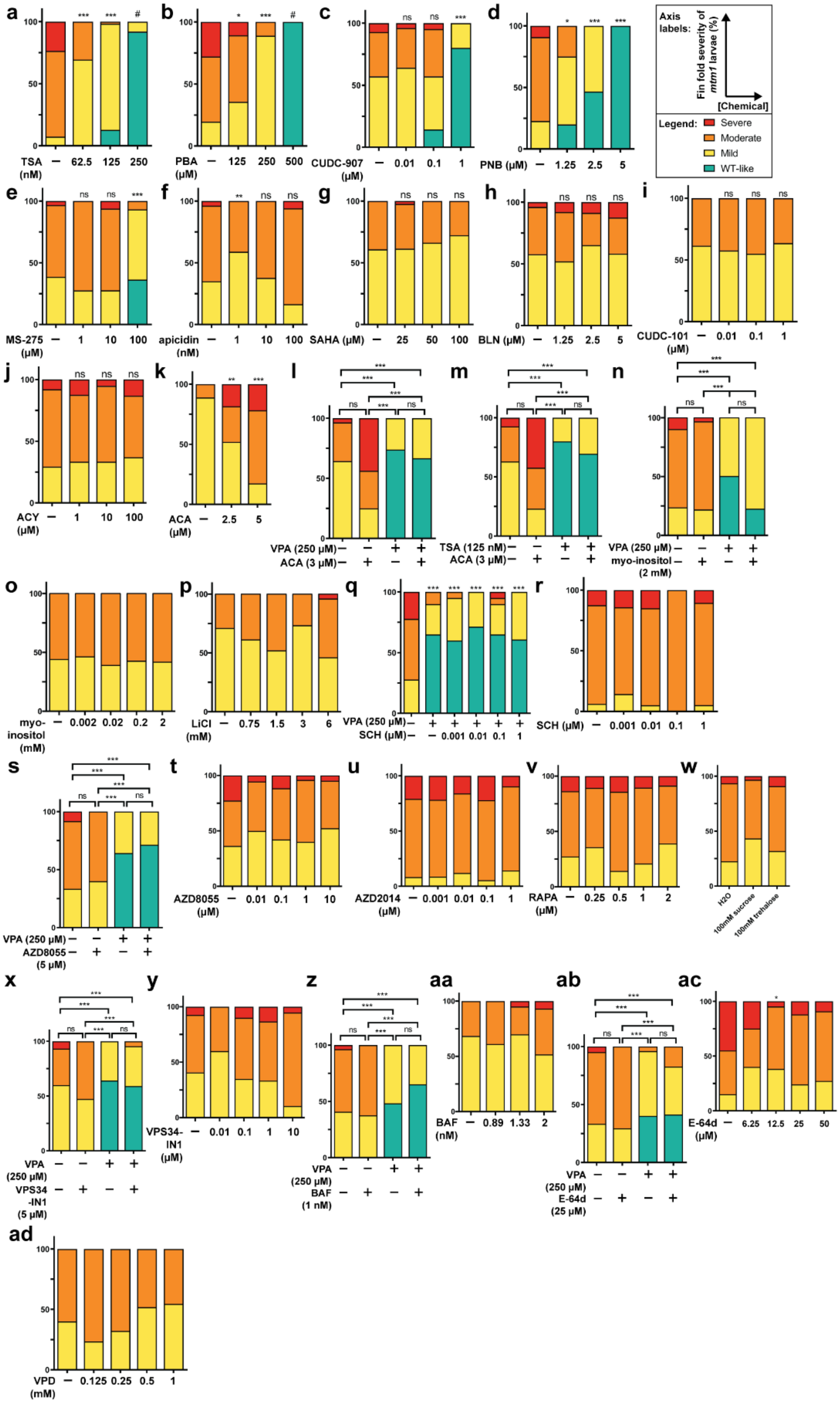

a
TSA (nM) — 62.5 125 250
b
PBA (µM) — 125 250 500
c
CUDC-907 (µM) — 0.01 0.1 1
d
PNB (µM) — 1.25 2.5 5
Axis labels: Fin fold severity of mtm1 larvae (%)
[Chemical]
Legend: Severe
Moderate
Mild
WT-like
e
MS-275 (µM) — 1 10 100
f
apicidin (nM) — 1 10 100
g
SAHA (µM) — 25 50 100
h
BLN (µM) — 1.25 2.5 5
i
CUDC-101 (µM) — 0.01 0.1 1
j
ACY (µM) — 1 10 100
k
ACA (µM) — 2.5 5
l
VPA (250 µM) — — + +
ACA (3 µM) — + — +
m
TSA (125 nM) — — + +
ACA (3 µM) — + — +
n
VPA (250 µM) — — + +
myo-inositol (2 mM) — + — +
o
myo-inositol (mM) — 0.002 0.02 0.2 2
p
LiCl (mM) — 0.75 1.5 3 6
q
VPA (250 µM) + + + + + +
SCH (µM) — — 0.001 0.01 0.1 1
r
SCH (µM) — 0.001 0.01 0.1 1
s
VPA (250 µM) — — + +
AZD8055 (5 µM) — + — +
t
AZD8055 (µM) — 0.01 0.1 1 10
u
AZD2014 (µM) — 0.001 0.01 0.1 1
v
RAPA (µM) — 0.25 0.5 1 2
w
H2O
100mM sucrose
100mM trehalose
x
VPA (250 µM) — — + +
VPS34-IN1 (5 µM) — + — +
y
VPS34-IN1 (µM) — 0.01 0.1 1 10
z
VPA (250 µM) — — + +
BAF (1 nM) — + — +
aa
BAF (nM) — 0.89 1.33 2
ab
VPA (250 µM) — — + +
E-64d (25 µM) — + — +
ac
E-64d (µM) — 6.25 12.5 25 50
ad
VPD (mM) — 0.125 0.25 0.5 1

Supplementary Fig. 2. Structurally dissimilar HDAC inhibitors suppress *mtm* fin degeneration, while other established VPA targets/pathways fail to affect the phenotype. A-E: Fin degeneration improves with exposure to escalating concentrations of **A:** trichostatin A (TSA) (*n*=55,62,62,62) #Note: 250 nM associated with developmental toxicity, **B:** 4-phenylbutyrate (PBA) (*n*=61,56,54,0) #Note: no mutants were identified at 500 μM concentration, **C:** CUDC-907 (*n*=14,25,21,15), **D:** panobinostat (PNB) (*n*=22,20,30,18) and **E:** MS-275 (n=62,47,65,44). Other HDAC inhibitors do not suppress *mtm* fin degeneration. Exposure to escalating concentrations of either **F:** apicidin (n=80,56,53,67), **G:** suberanilohydroxamic acid (SAHA) (*n*=46,44,65,58), **H:** belinostat (BLN) (n=26,25,23,24), **I:** CUDC-101 (*n*=26,26,29,22), or **J:** ricolinostat/ACY-1215 (ACY) (n=51,48,39,46) show no considerable effects on the *mtm* phenotype. **K:** Histone acetyltransferase inhibitor anacardic acid (ACA) worsens fin degeneration (n=27,27,23). **L-M:** HDAC inhibitors VPA (n=28,16,23,27) and TSA (n=27,26,25,23) suppress fin degeneration in the presence of ACA. **N-AC:** It is unlikely VPA acts via depletion of *myo*-inositol alone given that **N:** *myo*-inositol does not prevent suppression of fin degeneration by VPA (*n*=30,28,20,18), **O:** *myo*-inositol alone does not affect the fin phenotype (n=25,26,18,33,24), and **P:** treatment with lithium chloride (LiCl), also known to deplete *myo*-inositol, does not affect the fin phenotype (n=24,18,29,26,24). **Q-R:** It is unlikely VPA suppresses fin degeneration via activation of the ERK pathway alone given that **Q:** ERK1/2 inhibitor SCH772984 does not block VPA from suppressing fin degeneration (n=18,20,20,21,20,23) and **R:** SCH772984 (SCH) does not affect the fin phenotype (n=16,21,20,23,19). **S-AC:** It is unlikely that VPA suppresses fin degeneration by activating canonical PI3K-Akt-mTOR pathway alone, or otherwise modulating autophagy. **S:** mTOR inhibition with AZD8055 does not prevent phenotype suppression by VPA (*n*=24,30,28,21). Induction of autophagy with mTOR inhibitors **T:** rapamycin (RAPA) (n=22,28,21,19,23), **U:** AZD8055 (n=22,18,26,25,21), and **V:** AZD2014 (n=24,23,25,18,21) do not affect the fin phenotype. Nor does mTOR-independent induction of autophagy with **W:** trehalose (n=31,30,22). Disrupting autophagy via PIK3C3 inhibition with **X:** VPS34-IN1 (n=30,19,25,22), v-ATPase inhibition with **Z:** bafilomycin A1 (BAF) (n=27,24,29,23), or lysosomal cysteine protease inhibition with **AB:** E64d/aloxistatin (n=21,17,25,29) does not prevent suppression by VPA. Additionally, disrupting autophagy with **Y:** VPS34-IN1 (*n*=27,20,20,30,19), **AA:** bafilomycin A1 (n=19,18,20,29), or **AC:** E64d (n=20,20,21,25,22) does not affect fin degeneration. **AD:** A close structural analog of valproic acid, valpromide (VPD), which does not inhibit HDACs, does not suppress fin degeneration (n=25,17,28,27,22). Kruskal-Wallis test with Dunn's post-test.

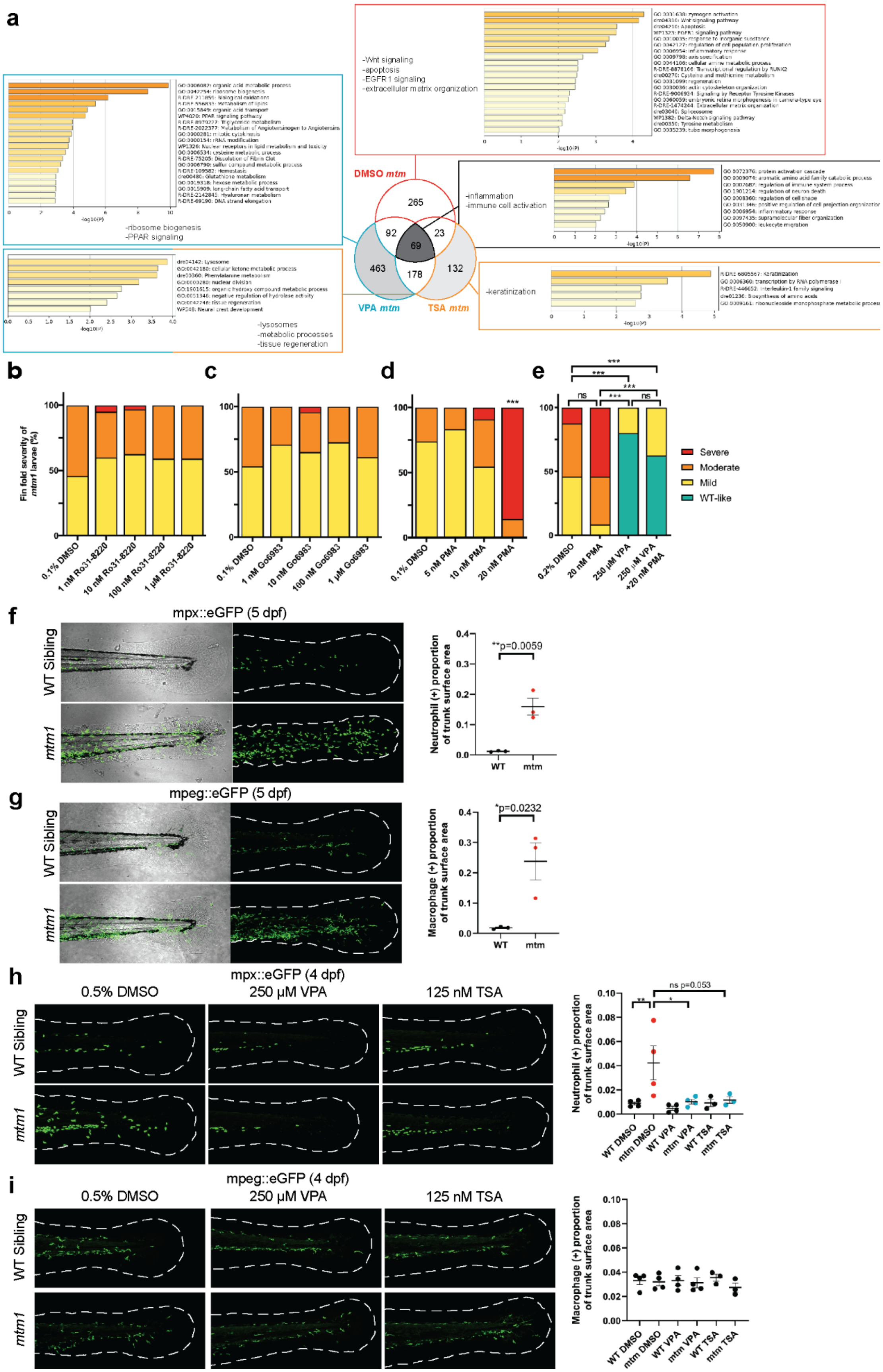
a
DMSO mtm
VPA mtm
TSA mtm
265
92
69
23
463
178
132
-Wnt signaling
-apoptosis
-EGFR1 signaling
-extracellular matrix organization
-ribosome biogenesis
-PPAR signaling
-inflammation
-immune cell activation
-lysosomes
-metabolic processes
-tissue regeneration
-keratinization
-log10(P)
b
c
d
e
Fin fold severity of mtm1 larvae (%)
0.1% DMSO
1 nM Ro31-8220
10 nM Ro31-8220
100 nM Ro31-8220
1 μM Ro31-8220
1 nM Go6983
10 nM Go6983
100 nM Go6983
1 μM Go6983
5 nM PMA
10 nM PMA
20 nM PMA
0.2% DMSO
250 μM VPA
250 μM VPA +20 nM PMA
Severe
Moderate
Mild
WT-like
ns
***
f
mpx::eGFP (5 dpf)
WT Sibling
mtm1
Neutrophil (+) proportion of trunk surface area
**p=0.0059
WT
mtm
g
mpeg::eGFP (5 dpf)
Macrophage (+) proportion of trunk surface area
*p=0.0232
h
mpx::eGFP (4 dpf)
0.5% DMSO
250 μM VPA
125 nM TSA
ns p=0.053
WT DMSO
mtm DMSO
WT VPA
mtm VPA
WT TSA
mtm TSA
i
mpeg::eGFP (4 dpf)

Supplementary Fig. 3. Comparative transcriptomics in *mtm* mutants treated with HDAC inhibitors. A: Venn diagram highlighting DEGs that are shared or unique to each group and their associated GO enrichment terms. **B-E:** To attempt to validate the transcriptional signature of PKC hyperactivity in mtm, several chemical modulators of PKC pathway were used. Exposure to pan-PKC inhibitors Ro31-8220 (**B,** n=24,20,32,22,22) or Go6983 (**C,** n=24,24,23,22,31) had no effect on the fin phenotype. **D:** Treatment of *mtm* with classical PKC activator phorbol 12-myristate 13-acetate (PMA) significantly worsened fin degeneration at low nanomolar concentrations (n=23,24,22,7). These data suggest that PKC pathways are not hyperactivated in *mtm*, but rather that converging downstream pathways generate the PKC hyperactivity transcriptional signature. **E:** Combination treatment with VPA negated this exacerbation consistent with VPA having PKC-independent action (n=24,20,24,24). Kruskal-Wallis test with Dunn's post-test. **F-I:** To understand why inflammatory and immune pathways are up-regulated in *mtm* mutants, we looked at neutrophil and macrophage distribution in *mtm* mutants using Tg(mpx::eGFP) (**F**) and Tg(mpeg::eGFP) (**G**) lines, respectively, and found increased expression of these cell populations in 5 dpf mutants. Unpaired, two-tailed T-test (n=3 each). **H-I:** At 4 dpf timepoint, neutrophils count is increased of which is normalized with HDAC inhibitor treatment. One-way ANOVA with Tukey's post-test (n=4 each).

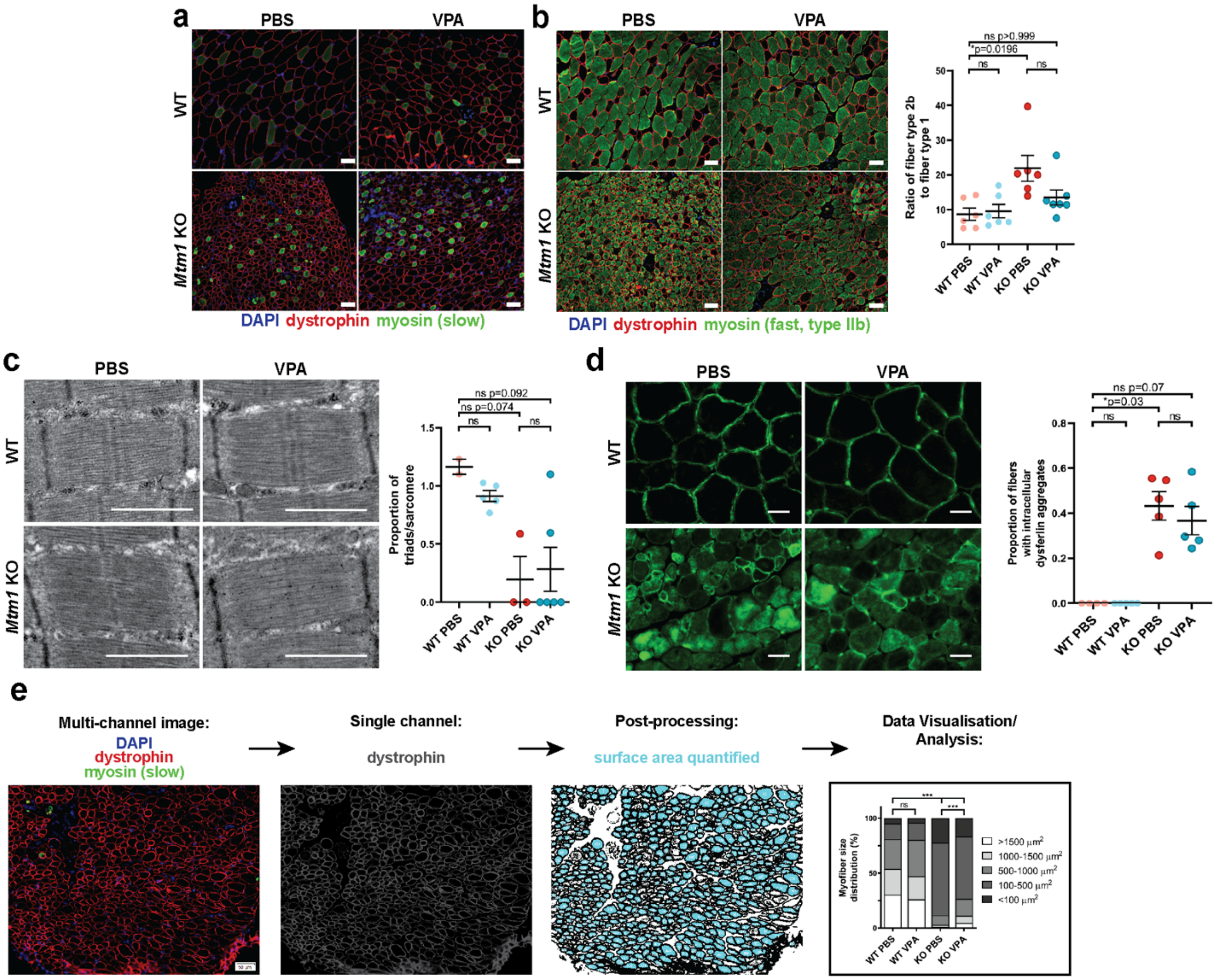

Supplementary Fig. 4. VPA does not improve certain defects in *Mtm* KO mice. A+B: Immunofluorescence staining for dystrophin (red), myosin heavy chain type 1 (slow, green) or type 2b (fast, green) with cross sections from tibialis anterior muscle tissue at 35 days of age. VPA improves the elevated ratio of type 2b to type 1 fibers. From left to right, Mean ratio of fiber type 2b to type 1 ±S.E.M are 8.706±1.724, 9.589±1.932, 21.89±3.739, 13.48±2.152, respectively; 7-10 different fields from n=6-7 mice; Kruskal-Wallis test with Dunn's post-test; scale bar = 50 µm. **C:** Electron microscopy of tibialis anterior muscle shows that VPA does not rescue triad defects. From left to right, the Mean proportion of triads/sarcomeres ± S.E.M are 1.165±0.065, 0.913±0.047, 0.196±0.196, 0.283±0.190, respectively; 3-4 different fields from n=2-6 mice; Kruskal-Wallis test with Dunn's post-test; scale bar = 1 µm. **D:** Dysferlin immunofluorescence shows that VPA does not reduce the prevalence of dysferlin aggregation in *Mtm1* KO fibers. From left to right, the proportion of fibers with intracellular aggregates ± S.E.M are 0±0, 0±0, 0.433±0.063, 0.368±0.063, respectively; eight different fields from n=4,5,5,5 mice; Kruskal-Wallis test with Dunn's post-test; scale bar = 20 µm**. E:** VPA treatment is associated with an increase in cross-sectional fiber area (µm2) as quantified using sections stained with dystrophin as a sarcolemmal membrane marker (n=6639, 5981,17672,11932 total fibers measured across 7-9 different sections from n=5-6 animals; Kruskal-Wallis test with Dunn's post-test). This quantification was performed using an automated macro in Fiji (see Methods) resulting in very rapid quantification of cross-sectional area of ~6x more fibers than was possible by manually measuring Feret's diameter. These data closely match that obtained using Feret's

diameter methodology (see FIG 3). The examples shown are from one field of a KO PBS sample. The majority of fibers are captured, but some are missed due to weak or fragmented staining around a fiber.

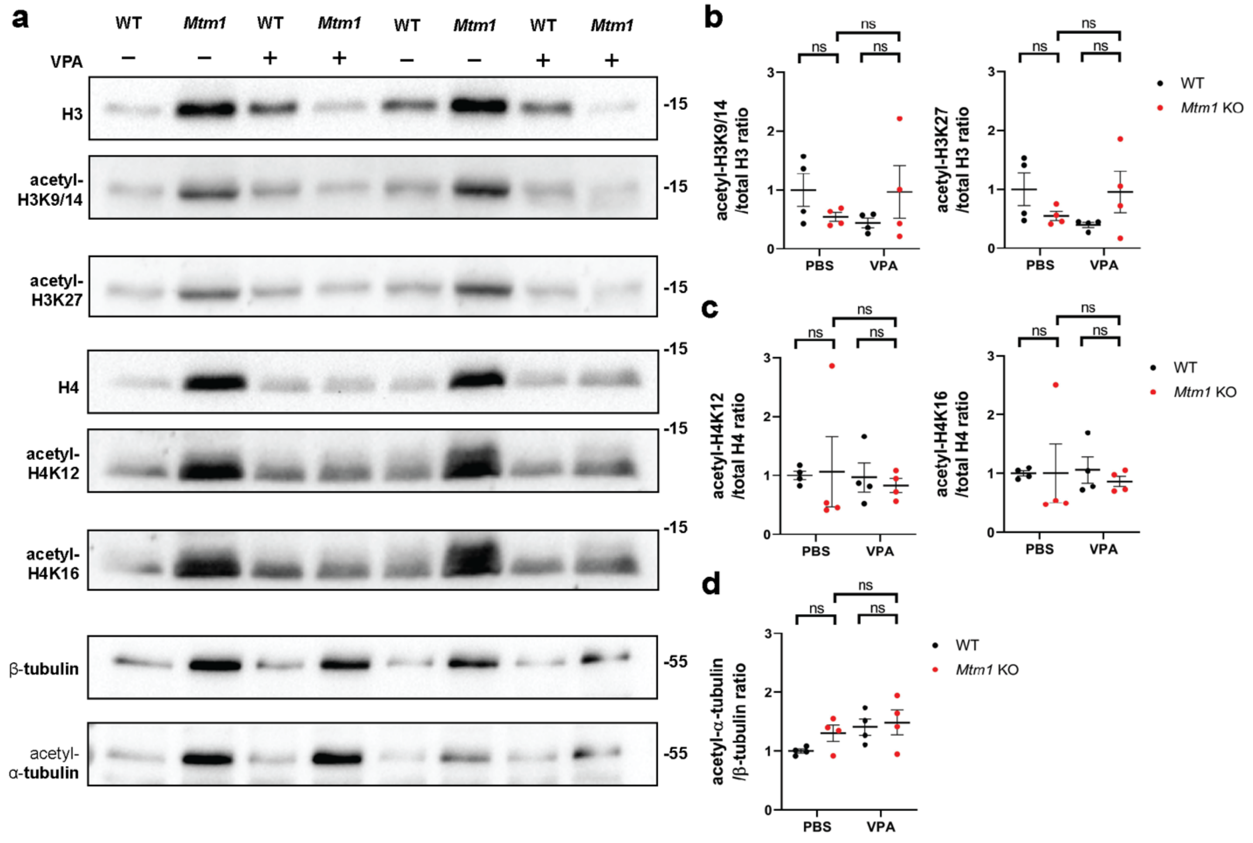

Supplementary Fig. 5. Effect of VPA on histone protein expression in *Mtm* KO mice A-D: VPA reduces the overall amount of histone proteins, which are elevated vs WT in untreated *Mtm1* KO mice. This is hypothesized to be a result of having more nuclei/same weight of tissue, given that fibers are considerably smaller in KO mice. VPA does not significantly increase the ratio of acetylated histones to total histone levels in KO skeletal muscle (Two-way ANOVA with Tukey's post-test; n=4 each)

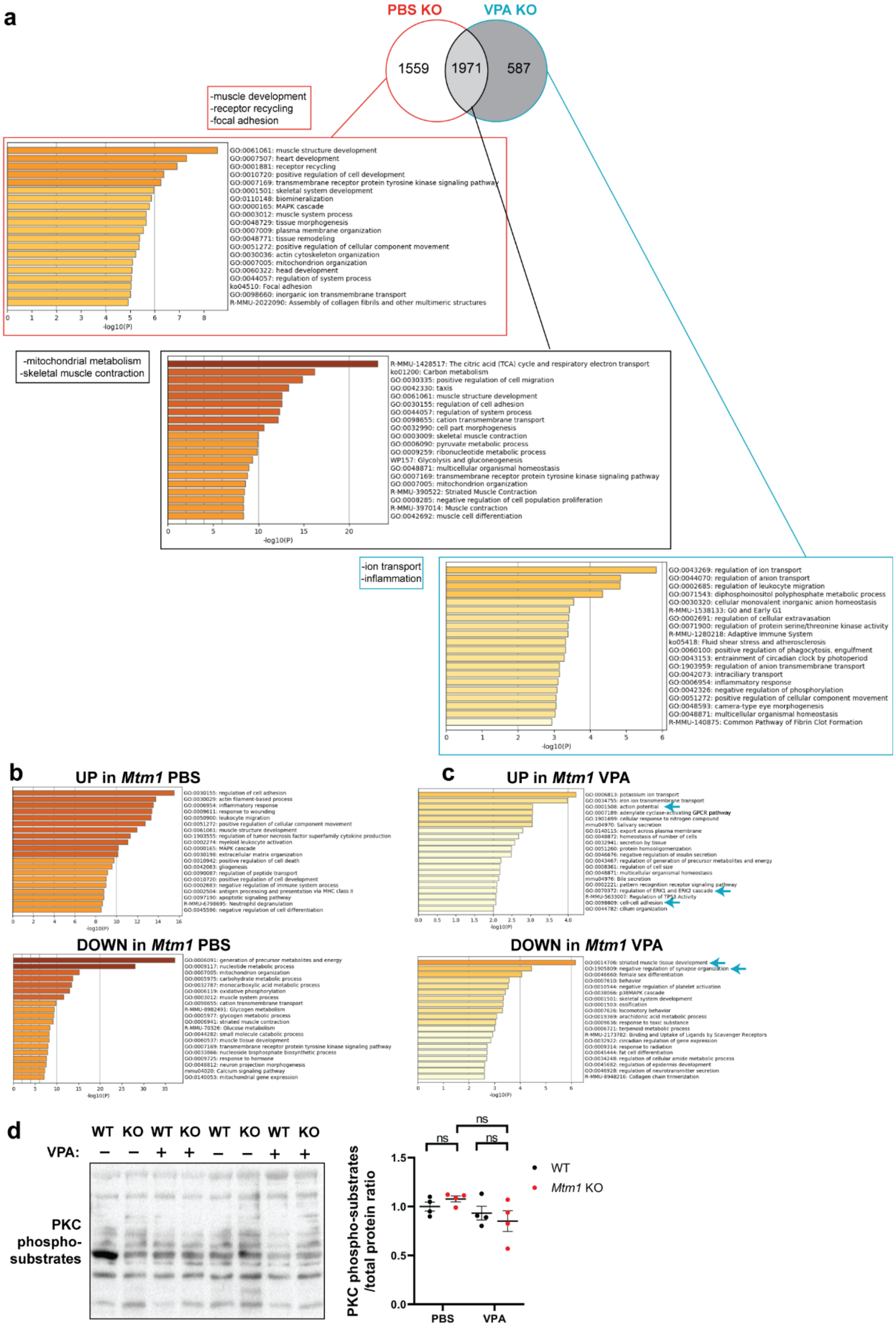
a
PBS KO
VPA KO
1559
1971
587
-muscle development
-receptor recycling
-focal adhesion
GO:0061061: muscle structure development
GO:0007507: heart development
GO:0001881: receptor recycling
GO:0010720: positive regulation of cell development
GO:0007169: transmembrane receptor protein tyrosine kinase signaling pathway
GO:0001501: skeletal system development
GO:0110148: biomineralization
GO:0000165: MAPK cascade
GO:0003012: muscle system process
GO:0048729: tissue morphogenesis
GO:0007009: plasma membrane organization
GO:0048771: tissue remodeling
GO:0051272: positive regulation of cellular component movement
GO:0030036: actin cytoskeleton organization
GO:0007005: mitochondrion organization
GO:0060322: head development
GO:0044057: regulation of system process
ko04510: Focal adhesion
GO:0098660: inorganic ion transmembrane transport
R-MMU-2022090: Assembly of collagen fibrils and other multimeric structures
-log10(P)
-mitochondrial metabolism
-skeletal muscle contraction
R-MMU-1428517: The citric acid (TCA) cycle and respiratory electron transport
ko01200: Carbon metabolism
GO:0030335: positive regulation of cell migration
GO:0042330: taxis
GO:0061061: muscle structure development
GO:0030155: regulation of cell adhesion
GO:0044057: regulation of system process
GO:0098655: cation transmembrane transport
GO:0032990: cell part morphogenesis
GO:0003009: skeletal muscle contraction
GO:0006090: pyruvate metabolic process
GO:0009259: ribonucleotide metabolic process
WP157: Glycolysis and gluconeogenesis
GO:0048871: multicellular organismal homeostasis
GO:0007169: transmembrane receptor protein tyrosine kinase signaling pathway
GO:0007005: mitochondrion organization
R-MMU-390522: Striated Muscle Contraction
GO:0008285: negative regulation of cell population proliferation
R-MMU-397014: Muscle contraction
GO:0042692: muscle cell differentiation
-log10(P)
-ion transport
-inflammation
GO:0043269: regulation of ion transport
GO:0044070: regulation of anion transport
GO:0002685: regulation of leukocyte migration
GO:0071543: diphosphoinositol polyphosphate metabolic process
GO:0030320: cellular monovalent inorganic anion homeostasis
R-MMU-1538133: G0 and Early G1
GO:0002691: regulation of cellular extravasation
GO:0071900: regulation of protein serine/threonine kinase activity
R-MMU-1280218: Adaptive Immune System
ko05418: Fluid shear stress and atherosclerosis
GO:0060100: positive regulation of phagocytosis, engulfment
GO:0043153: entrainment of circadian clock by photoperiod
GO:1903959: regulation of anion transmembrane transport
GO:0042073: intraciliary transport
GO:0006954: inflammatory response
GO:0042326: negative regulation of phosphorylation
GO:0051272: positive regulation of cellular component movement
GO:0048593: camera-type eye morphogenesis
GO:0048871: multicellular organismal homeostasis
R-MMU-140875: Common Pathway of Fibrin Clot Formation
-log10(P)
b
UP in Mtm1 PBS
DOWN in Mtm1 PBS
c
UP in Mtm1 VPA
DOWN in Mtm1 VPA
d
WT KO WT KO WT KO WT KO
VPA: – – + + – – + +
PKC phospho-substrates
PKC phospho-substrates /total protein ratio
ns
ns
ns
PBS
VPA
WT
Mtm1 KO

Supplementary Fig. 6. Comparative transcriptomics in *Mtm1* KO mice treated with VPA. A: Venn diagram highlighting DEGs that are shared or unique to each *Mtm1* KO treatment compared to WT PBS and their associated GO enrichment terms. **B-C:** GO term enrichment separated by up- and down-regulated DEGs, showing correction of specific transcriptional pathways. **D:** In attempts to validate the transcriptional signature of PKC hyperactivity in *Mtm1*, we probed for phosphorylated PKC substrates. We observed a non-significant trend of decreasing levels of phospho-PKC substrates after VPA treatment (Two-way ANOVA with Tukey's post-test; *n*=4 each).

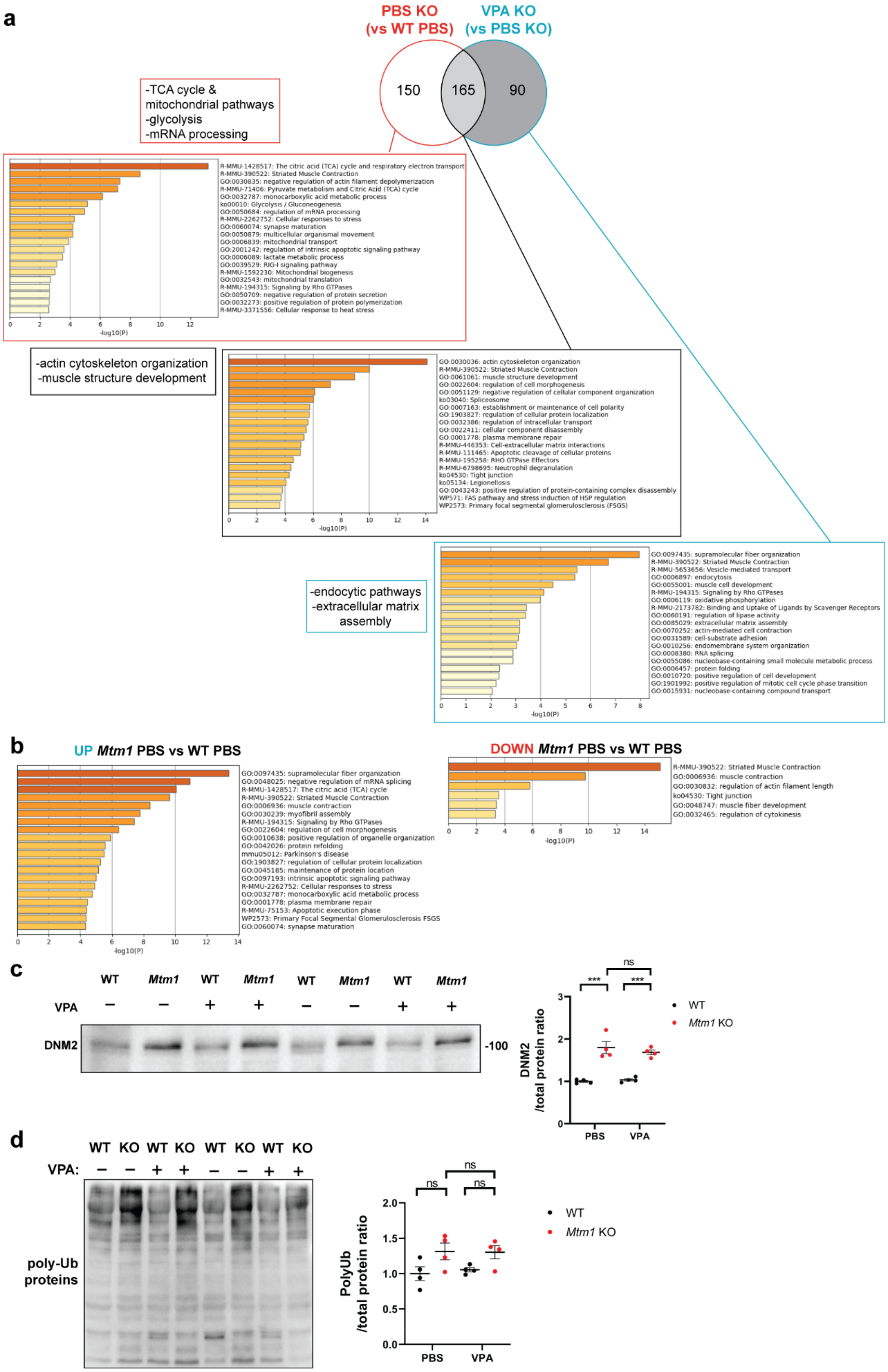
a
PBS KO (vs WT PBS)
VPA KO (vs PBS KO)
150
165
90
-TCA cycle & mitochondrial pathways
-glycolysis
-mRNA processing
R-MMU-1428517: The citric acid (TCA) cycle and respiratory electron transport
R-MMU-390522: Striated Muscle Contraction
GO:0030835: negative regulation of actin filament depolymerization
R-MMU-71406: Pyruvate metabolism and Citric Acid (TCA) cycle
GO:0032787: monocarboxylic acid metabolic process
ko00010: Glycolysis / Gluconeogenesis
GO:0050684: regulation of mRNA processing
R-MMU-2262752: Cellular responses to stress
GO:0060074: synapse maturation
GO:0050879: multicellular organismal movement
GO:0006839: mitochondrial transport
GO:2001242: regulation of intrinsic apoptotic signaling pathway
GO:0006089: lactate metabolic process
GO:0039529: RIG-I signaling pathway
R-MMU-1592230: Mitochondrial biogenesis
GO:0032543: mitochondrial translation
R-MMU-194315: Signaling by Rho GTPases
GO:0050709: negative regulation of protein secretion
GO:0032273: positive regulation of protein polymerization
R-MMU-3371556: Cellular response to heat stress
-log10(P)
-actin cytoskeleton organization
-muscle structure development
GO:0030036: actin cytoskeleton organization
R-MMU-390522: Striated Muscle Contraction
GO:0061061: muscle structure development
GO:0022604: regulation of cell morphogenesis
GO:0051129: negative regulation of cellular component organization
ko03040: Spliceosome
GO:0007163: establishment or maintenance of cell polarity
GO:1903827: regulation of cellular protein localization
GO:0032386: regulation of intracellular transport
GO:0022411: cellular component disassembly
GO:0001778: plasma membrane repair
R-MMU-446353: Cell-extracellular matrix interactions
R-MMU-111465: Apoptotic cleavage of cellular proteins
R-MMU-195258: RHO GTPase Effectors
R-MMU-6798695: Neutrophil degranulation
ko04530: Tight junction
ko05134: Legionellosis
GO:0043243: positive regulation of protein-containing complex disassembly
WP571: FAS pathway and stress induction of HSP regulation
WP2573: Primary focal segmental glomerulosclerosis (FSGS)
-log10(P)
-endocytic pathways
-extracellular matrix assembly
GO:0097435: supramolecular fiber organization
R-MMU-390522: Striated Muscle Contraction
R-MMU-5653656: Vesicle-mediated transport
GO:0006897: endocytosis
GO:0055001: muscle cell development
R-MMU-194315: Signaling by Rho GTPases
GO:0006119: oxidative phosphorylation
R-MMU-2173782: Binding and Uptake of Ligands by Scavenger Receptors
GO:0060191: regulation of lipase activity
GO:0085029: extracellular matrix assembly
GO:0070252: actin-mediated cell contraction
GO:0031589: cell-substrate adhesion
GO:0010256: endomembrane system organization
GO:0008380: RNA splicing
GO:0055086: nucleobase-containing small molecule metabolic process
GO:0006457: protein folding
GO:0010720: positive regulation of cell development
GO:1901992: positive regulation of mitotic cell cycle phase transition
GO:0015931: nucleobase-containing compound transport
-log10(P)
b
UP Mtm1 PBS vs WT PBS
GO:0097435: supramolecular fiber organization
GO:0048025: negative regulation of mRNA splicing
R-MMU-1428517: The citric acid (TCA) cycle
R-MMU-390522: Striated Muscle Contraction
GO:0006936: muscle contraction
GO:0030239: myofibril assembly
R-MMU-194315: Signaling by Rho GTPases
GO:0022604: regulation of cell morphogenesis
GO:0010638: positive regulation of organelle organization
GO:0042026: protein refolding
mmu05012: Parkinson's disease
GO:1903827: regulation of cellular protein localization
GO:0045185: maintenance of protein location
GO:0097193: intrinsic apoptotic signaling pathway
R-MMU-2262752: Cellular responses to stress
GO:0032787: monocarboxylic acid metabolic process
GO:0001778: plasma membrane repair
R-MMU-75153: Apoptotic execution phase
WP2573: Primary Focal Segmental Glomerulosclerosis FSGS
GO:0060074: synapse maturation
-log10(P)
DOWN Mtm1 PBS vs WT PBS
R-MMU-390522: Striated Muscle Contraction
GO:0006936: muscle contraction
GO:0030832: regulation of actin filament length
ko04530: Tight junction
GO:0048747: muscle fiber development
GO:0032465: regulation of cytokinesis
-log10(P)
c
WT Mtm1 WT Mtm1 WT Mtm1 WT Mtm1
VPA − − + + − − + +
DNM2
-100
DNM2 /total protein ratio
ns
***
***
WT
Mtm1 KO
PBS
VPA
d
WT KO WT KO WT KO WT KO
VPA: − − + + − − + +
poly-Ub proteins
PolyUb /total protein ratio
ns
ns
ns
WT
Mtm1 KO
PBS
VPA

Supplementary Fig. 7. Protein changes in *Mtm1* KO mice treated with VPA. A: Venn diagram highlighting DEPs (as derived from comparative proteomics of skeletal muscle extracts) that are shared or unique to PBS treated *Mtm1* KOs along with selected associated GO enrichment terms. **B:** Enriched GO terms in PBS treated *Mtm1* KO vs WT separated by up- and down-regulated DEPs. **C:** Western blot showing that VPA treatment does not reduce elevated levels of DNM2 in *Mtm1* KOs (Two-way ANOVA with Tukey's post-test; n=4 each). **D:** Western blot showing that VPA does not reduce elevated levels of poly-ubiquitinated (Ub) proteins in *Mtm1* KOs (Two-way ANOVA with Tukey's post-test; n=4 each).

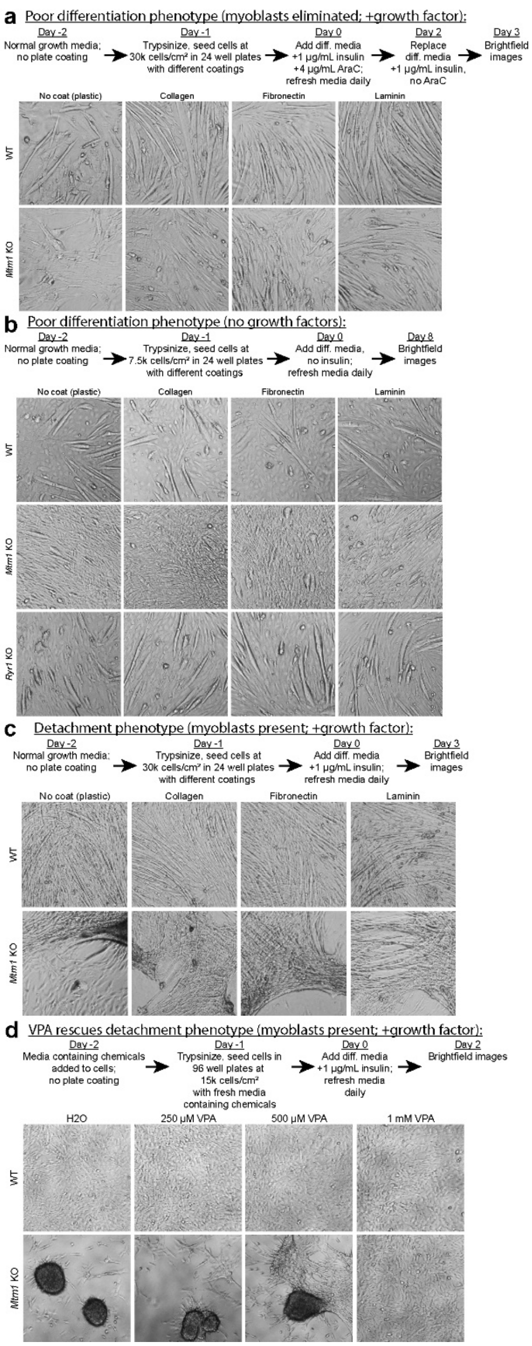

Supplementary Fig. 8. *Mtm1* C2C12 KO cells show abnormal differentiation phenotypes. A: Under conditions where myoblasts are eliminated (using AraC) post differentiation induction, KO fibers do not differentiate as well as WT fibers. Laminin coating appears to support the best differentiation of *Mtm1* fibers under these conditions. **B:** Under standard differentiation conditions (where myoblasts are not eliminated), differentiation of KO fibers is significantly altered as compared to WT or *Ryr1* KO C2C12 cells. **C:** Under standard differentiation conditions with insulin added as a growth factor, differentiating KO myoblasts clump up and detach from culture dishes. **D:** Exposure to VPA reduces the number of clumps and extent of detachment, fully suppressing this phenotype at 1 mM.

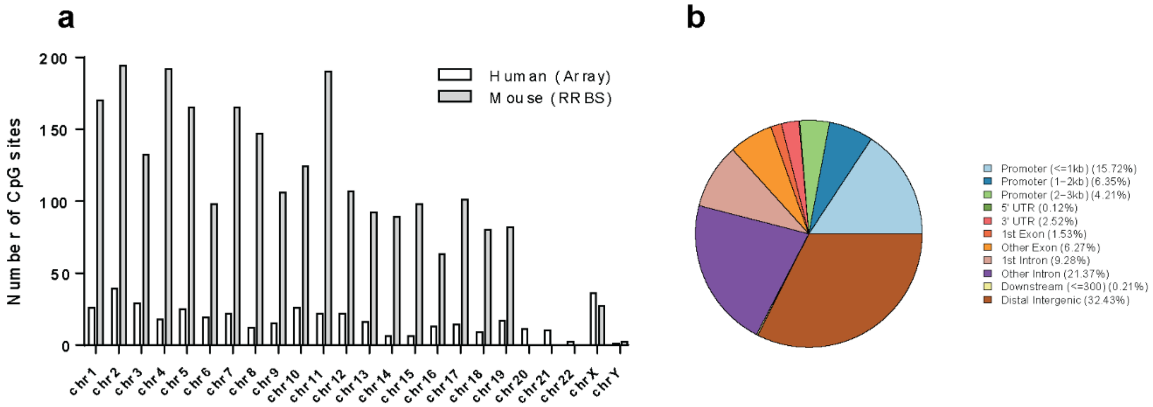

Supplementary Fig. 9: Chromosome distribution and differentially methylated regions in XLMTM patients and *Mtm1* KO mice. A: Gene mapping shows that differentially methylated (DM) CpGs are distributed throughout the genome in both XLMTM patients and *Mtm1* KO mice. **B:** Pie-chart showing genomic region mapping of CpGs. The largest fractions of DM regions mapped to either introns or intergenic regions, while the remaining DM CpGs mapped to various features in proximal gene regions, including promoters, 5' UTRs, and first exons.
